# Supplementary material for: Bridging cancer cell-intrinsic driver genes and -extrinsic cell-cell communication with Driver2Comm
Source: PLoS Comput Biol. 2026 Feb 17;22(2):e1013973. doi: 10.1371/journal.pcbi.1013973 (PMC12928580; doi:10.1371/journal.pcbi.1013973)
Supplement: S1 Text — (DOCX) [file pcbi.1013973.s001.docx]

Supplementary Information

Supplementary Figures and Tables

Fig A. Visualization of CNV profile of the PDAC dataset.


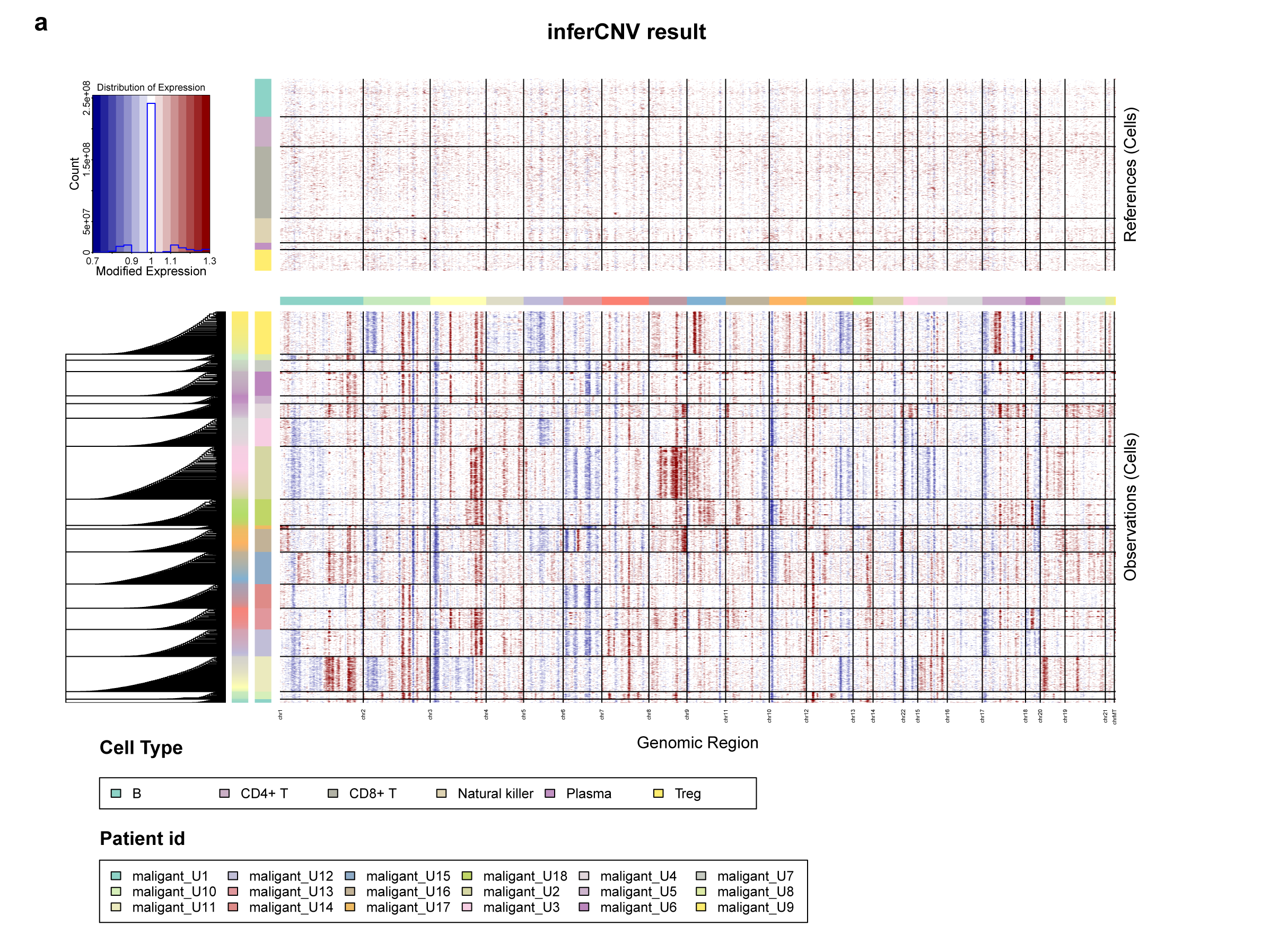


**Fig A.** **Visualization of CNV profile of the PDAC dataset. a** CNV profile in cancer versus references diploid cells assessed using InferCNV on the PDAC dataset.

Fig B. Visualization of driver-associated CCC signatures in the PDAC dataset.


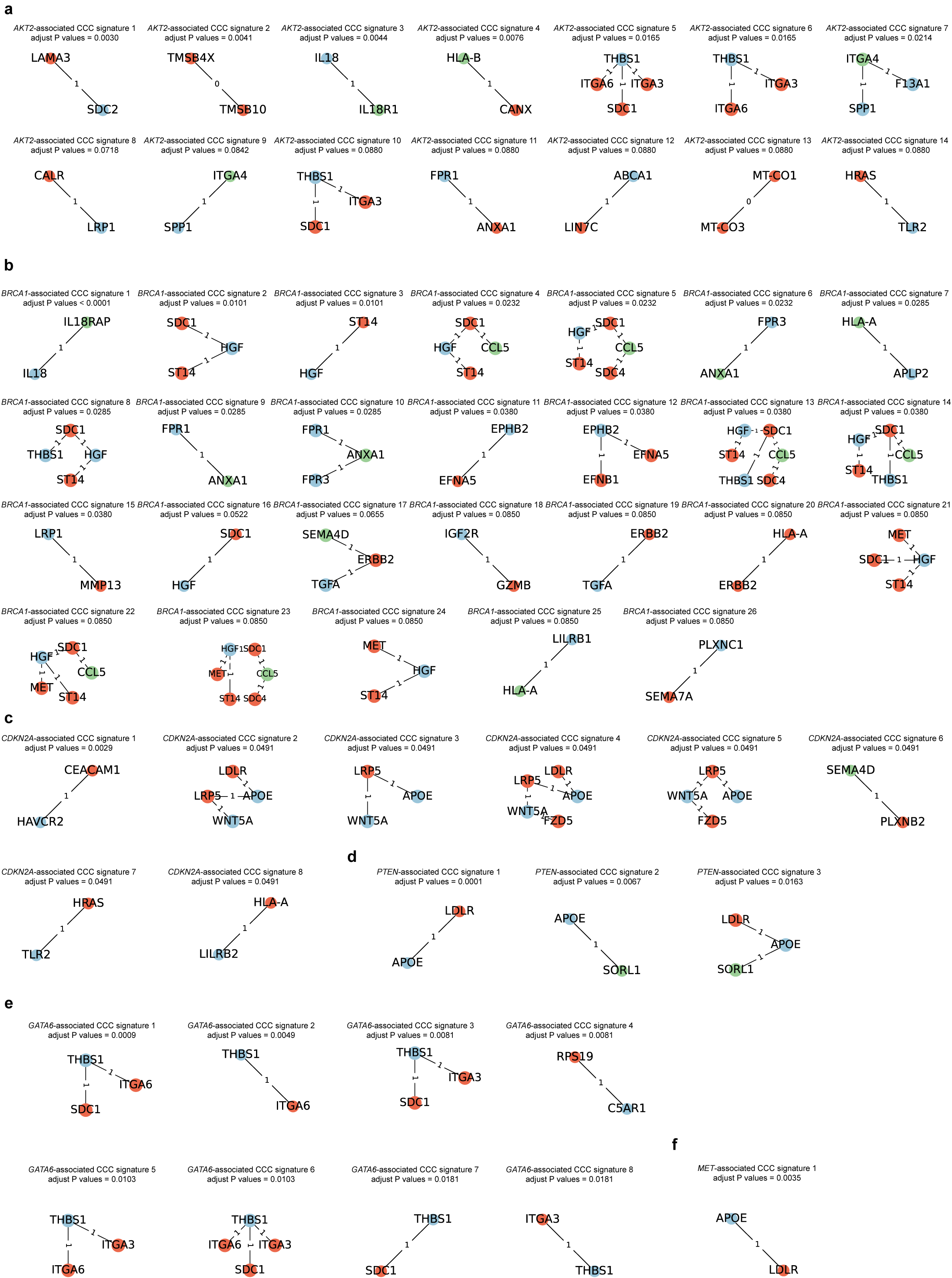


**Fig B.** **Visualization of driver-associated CCC signatures in the PDAC dataset.** (**a-f**) CCC signatures significantly associated with *AKT2* (**a**), *BRCA1* (**b**), *CDKN2A* (**c**), *PTEN* (**d**), *GATA6* (**e**), and *MET* (**f**). *P* values of cancer driver-associated CCC signatures were computed using the Fisher’s exact test and adjusted using the method of Benjamini-Hochberg procedure. All CCC signatures were ranked according to their adjusted *P* values. Node labels are gene names and different colors are used to distinguish cell types with red for tumor cells, blue for macrophages, and green for CD8+ T cells. Edge labels represent different edge types with “0” for intracellular edges and “1” for intercellular edges. TNBC, Triple Negative Breast Cancer.

Fig C. IE pathway visualization of top 10 CCC signatures associated with *BRCA1* in the PDAC dataset.


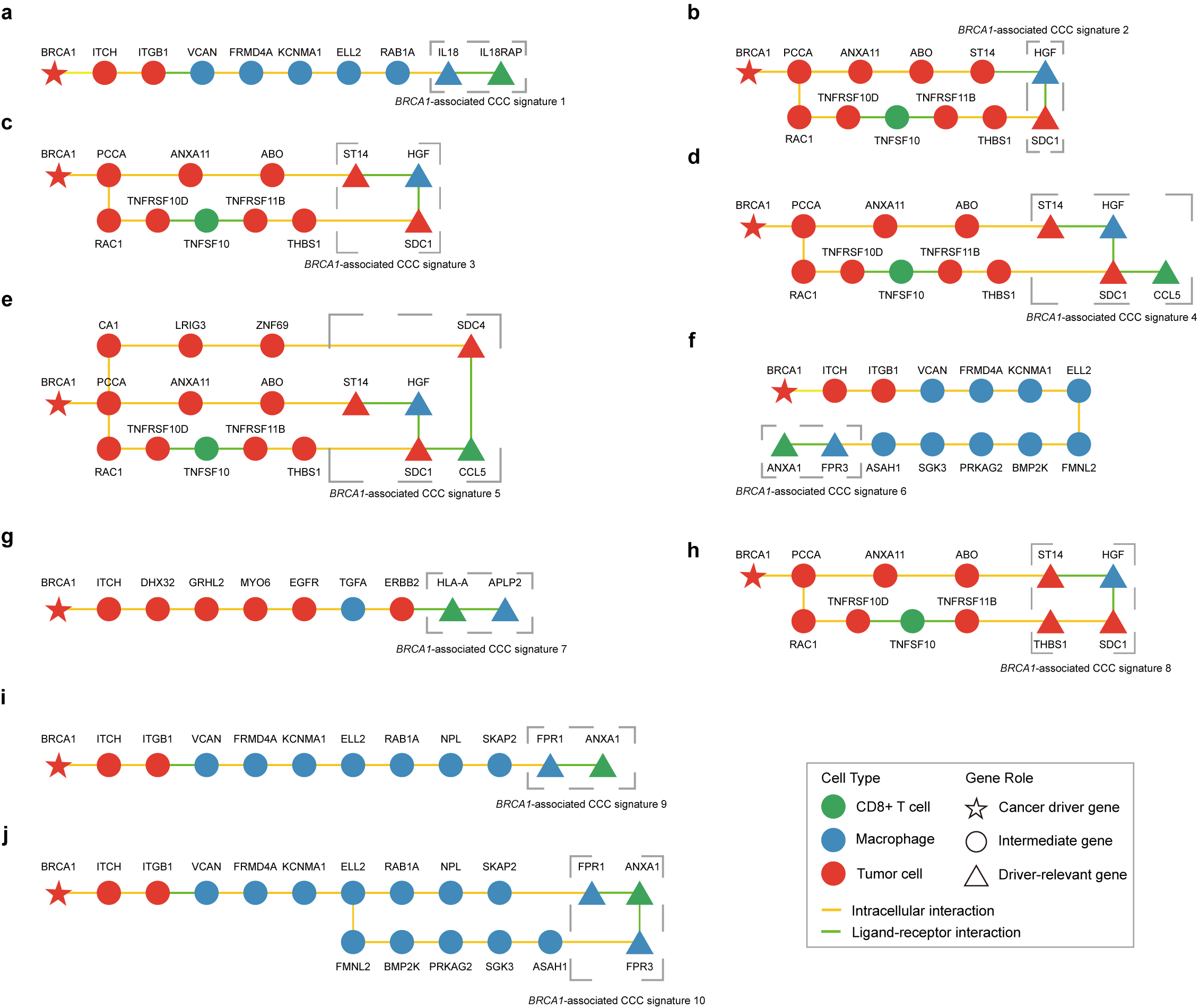


**Fig C.** **IE pathway visualization of top 10 CCC signatures associated with *BRCA1* in the PDAC dataset. a-j** The IE pathways between *BRCA1* and the top 10 *BRCA1*-associated CCC signatures in the MCTC network.

Fig D. IE pathway visualization of top 10 CCC signatures associated with *AKT2* in the PDAC dataset.


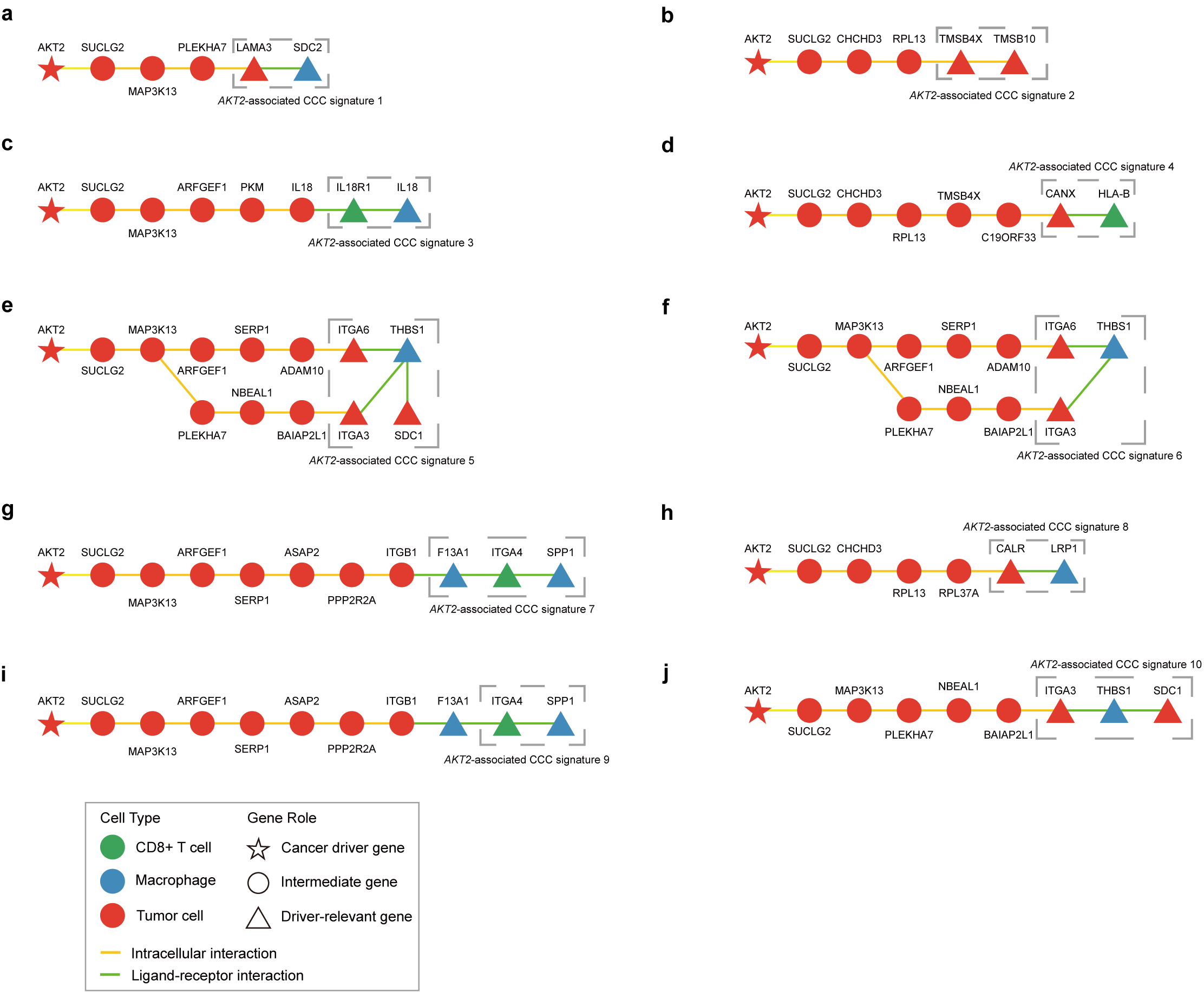


**Fig D.** **IE pathway visualization of top 10 CCC signatures associated with *AKT2* in the PDAC dataset. a-j** The IE pathways between *AKT2* and the top 10 *AKT2*-associated CCC signatures in the MCTC network.

Fig E. Flow diagram of prognostic evaluation using deconvolution of bulk sequencing data.


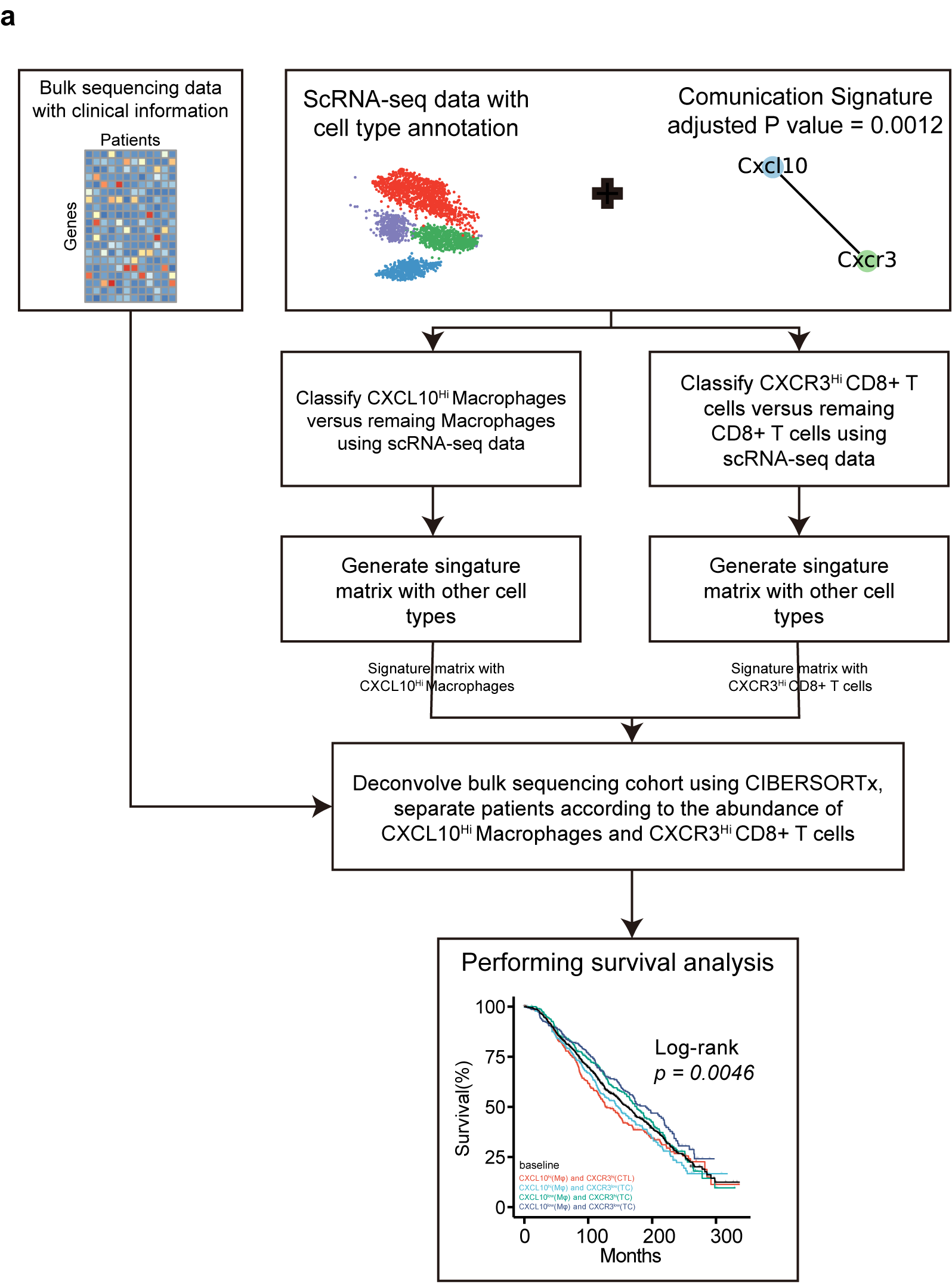


**Fig E. Flow diagram of prognostic evaluation using deconvolution of bulk sequencing data. a** Prognostic evaluation step in this study required bulk sequencing data with clinical information, scRNA-seq data with cell type annotation, and CCC signatures significantly associated with cancer driver or tumor subtype as input. Classification of cell type and separation of patients were both stratified by their median values. Mφ, Macrophage; CTL, CD8+ T cell.

Fig F. Survival analysis of all significant *BRCA1*-associated CCC signatures in PDAC TCGA patient cohort.


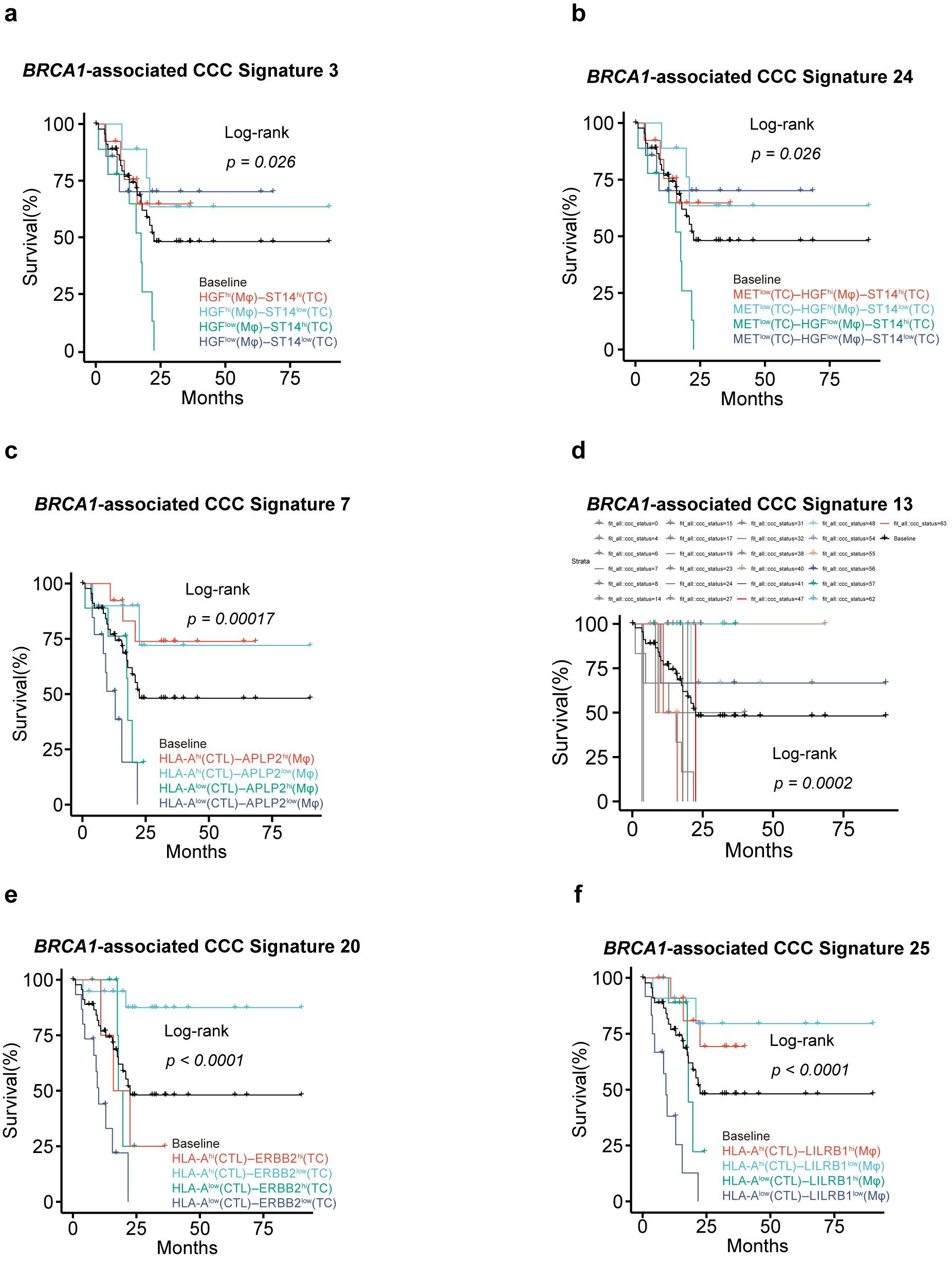


**Fig F.** **Survival analysis of all significant *BRCA1*-associated CCC signatures in PDAC TCGA patient cohort.** **a-f** Kaplan-Meier analysis of overall survival outcomes in patients with *BRCA1* variation in TCGA (n = 46 patients in total) with various expression levels of all significant *BRCA1*-associated CCC signatures. Baseline curve represents the overall clinical outcome of all PDAC TCGA patients with *BRCA1* variation in METABRIC. Patients were stratified according to the expression of genes involved in driver gene-associated CCC signatures. For each gene in a given CCC signature, patients were categorized into highly-expressed and lowly-expressed groups based on the median expression value. All possible combinations of high/low states across the involved genes in the CCC signatures were considered, and each colored curve represents one such combinatorial expression pattern, as indicated in the legend. Survival differences among all stratified groups were evaluated using a multi-group log-rank test, and the reported *P* values correspond to the global comparison across all groups within each panel. CCC, cell-cell communication; TC, Tumor Cell; CTL, CD8+ T cell; Mφ, Macrophage.

Fig G. Survival analysis of all significant *AKT2*-associated CCC signatures in PDAC TCGA patient cohort.


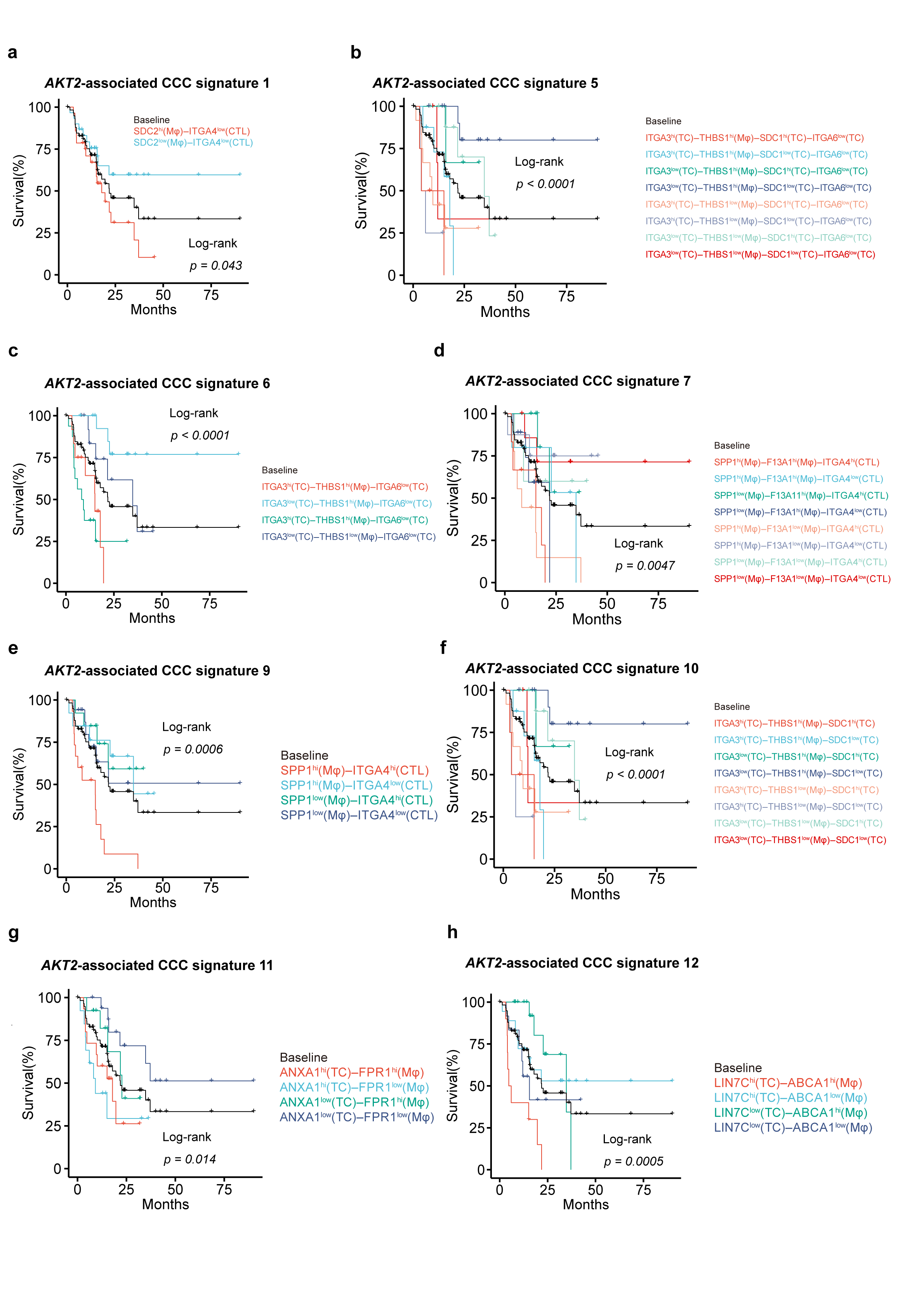


**Fig G.** **Survival analysis of all significant *AKT2*-associated CCC signatures in PDAC TCGA patient cohort.** **a-h** Kaplan-Meier analysis of overall survival outcomes in patients with *AKT2* variation in TCGA (n = 59 patients in total) with various expression levels of all significant *AKT2*-associated CCC signatures. All P values were computed by Log-rank Test. Baseline curve represents the overall clinical outcome of all PDAC TCGA patients with *AKT2* variation in METABRIC. Patients were stratified according to the expression of genes involved in driver gene-associated CCC signatures. For each gene in a given CCC signature, patients were categorized into highly-expressed and lowly-expressed groups based on the median expression value. All possible combinations of high/low states across the involved genes in the CCC signatures were considered, and each colored curve represents one such combinatorial expression pattern, as indicated in the legend. Survival differences among all stratified groups were evaluated using a multi-group log-rank test, and the reported *P* values correspond to the global comparison across all groups within each panel. CCC, cell-cell communication; TC, Tumor Cell; CTL, CD8+ T cell; Mφ, Macrophage.

Fig H. Visualization of CCC signatures associated with *ESR1*, *ERBB2*, and TNBC in breast cancer dataset from Wu et al..


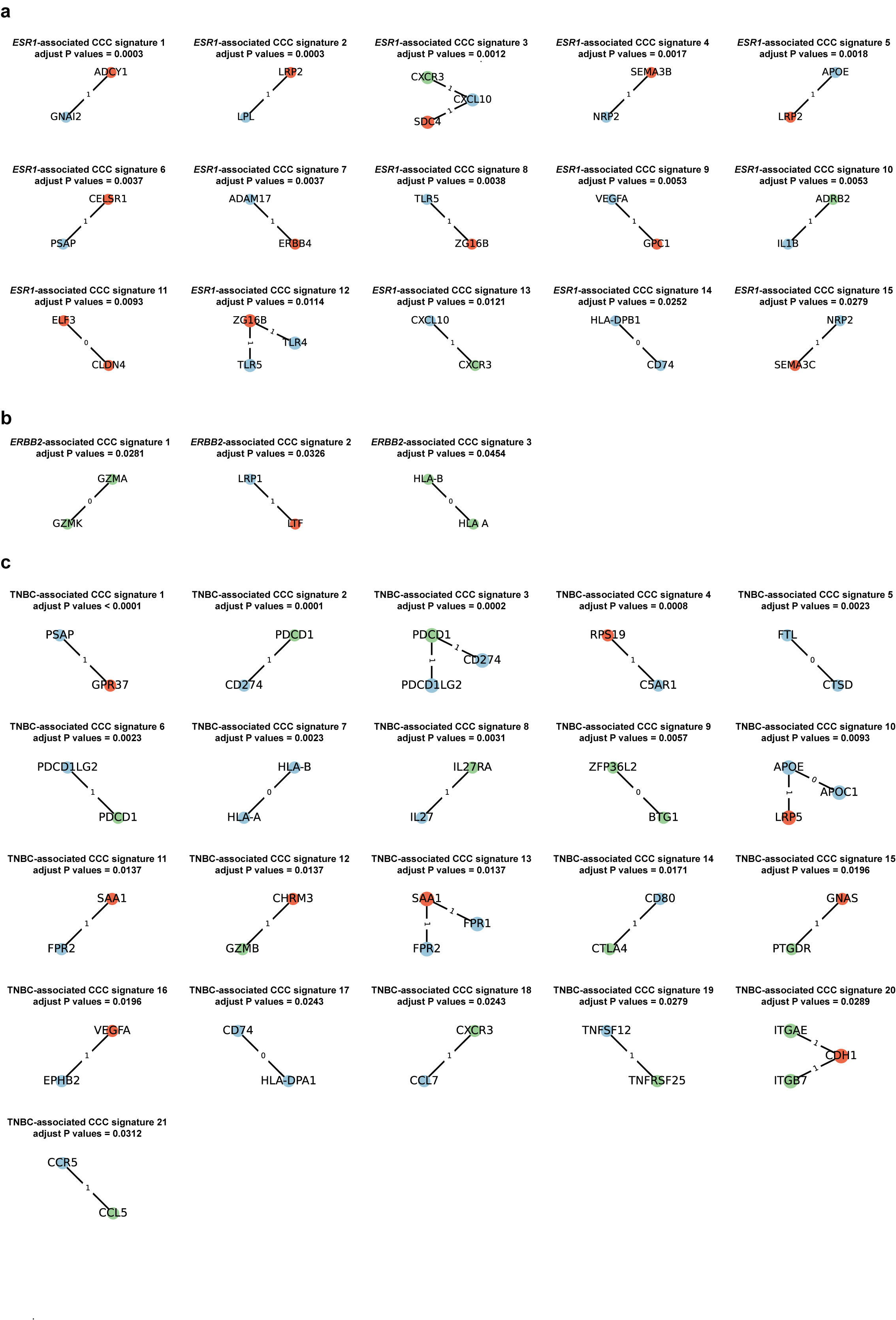


**Fig H.** **Visualization of CCC signatures associated with *ESR1*, *ERBB2*, and TNBC in breast cancer dataset from Wu et al..** **a-c** CCC signatures significantly associated with *ESR1*(**a**), *ERBB2*(**b**), and TNBC(**c**). *P* values of cancer driver-associated CCC signatures were computed using the Fisher’s exact test and adjusted using the method of Benjamini-Hochberg procedure. All CCC signatures were ranked according to their adjusted *P* values. Node labels are gene names and different colors are used to distinguish cell types with red for tumor cells, blue for macrophages, and green for CD8+ T cells. Edge labels represent different edge types with “0” for intracellular edges and “1” for intercellular edges. TNBC, Triple Negative Breast Cancer.

Fig I. IE pathway visualization of top 10 CCC signatures associated with *ESR1* in breast cancer dataset from Wu et al..


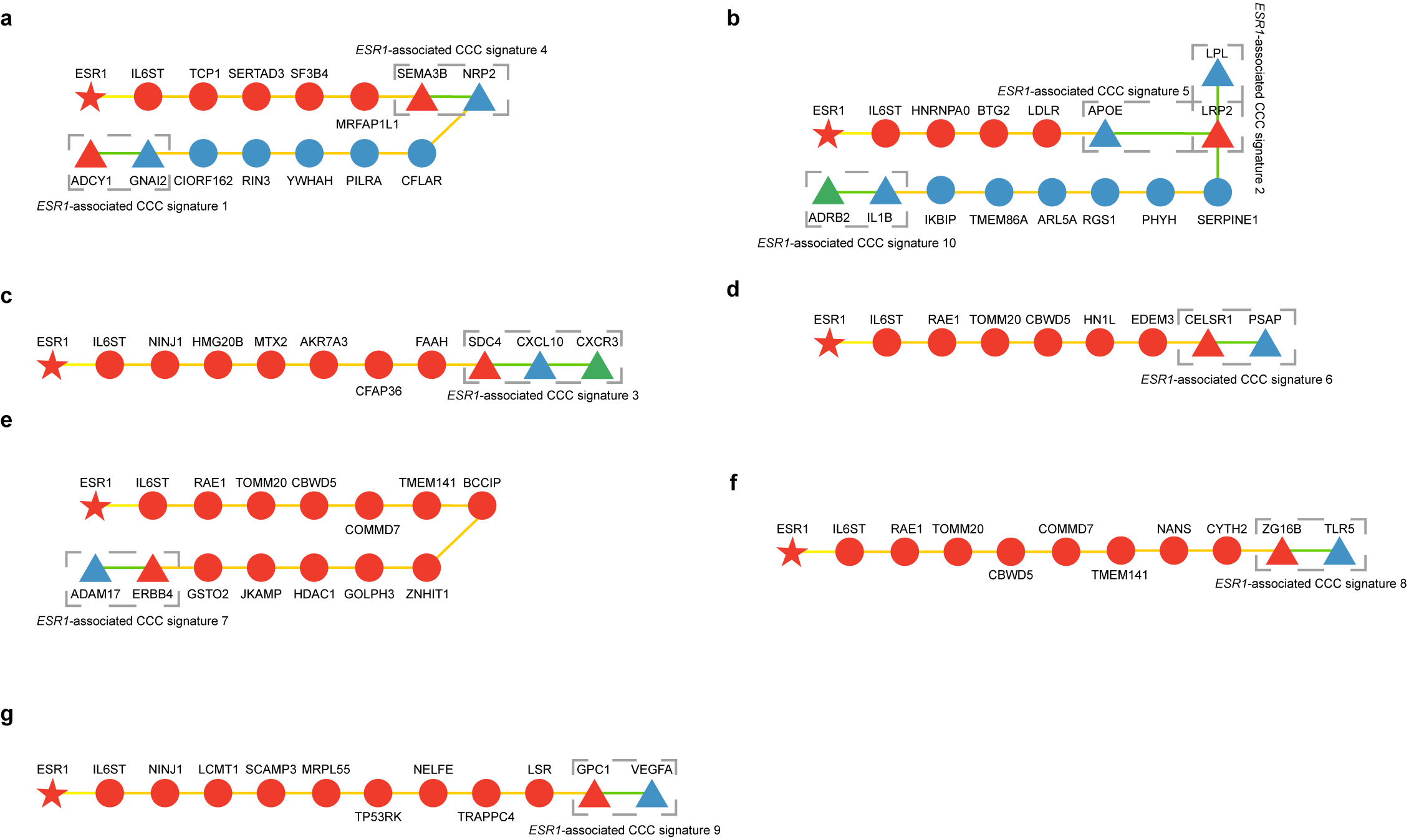


**Fig I.** **IE pathway visualization of top 10 CCC signatures associated with *ESR1* in breast cancer** **dataset from Wu et al.. a-g** The IE pathways between *ESR1* and the top 10 *ESR1*-associated CCC signatures in the MCTC network.

Fig J. Survival analysis of all significant *ESR1*-associated CCC signatures in ER+ METABRIC patient cohort.


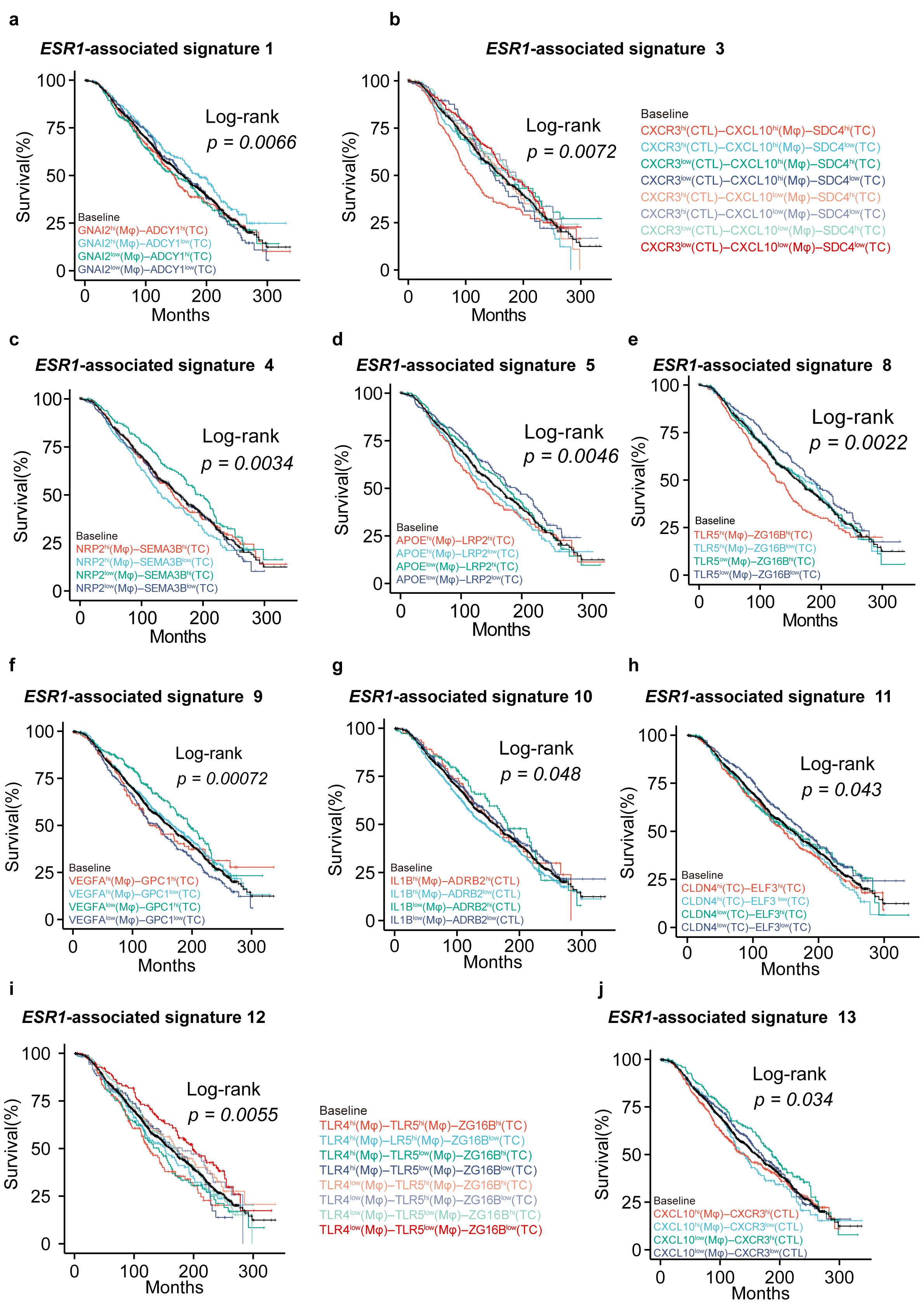


**Fig J. Survival analysis of all significant *ESR1*-associated CCC signatures in ER+ METABRIC patient cohort.** **a-j** Kaplan-Meier analysis of overall survival outcomes in patients in METABRIC (n = 1196 patients in total) with various expression levels of all significant *ESR1*-associated CCC signatures. All *P* values were computed by Log-rank Test. Baseline curve represents the overall clinical outcome of all ER+ patients in METABRIC. Patients were stratified according to the expression of genes involved in driver gene-associated CCC signatures. For each gene in a given CCC signature, patients were categorized into highly-expressed and lowly-expressed groups based on the median expression value. All possible combinations of high/low states across the involved genes in the CCC signatures were considered, and each colored curve represents one such combinatorial expression pattern, as indicated in the legend. Survival differences among all stratified groups were evaluated using a multi-group log-rank test, and the reported *P* values correspond to the global comparison across all groups within each panel. CCC, cell-cell communication; TC, Tumor Cell; CTL, CD8+ T cell; Mφ, Macrophage.

Fig K. Survival analysis of genes in all *ESR1*-associated CCC signatures in ER+ METABRIC patient cohort.


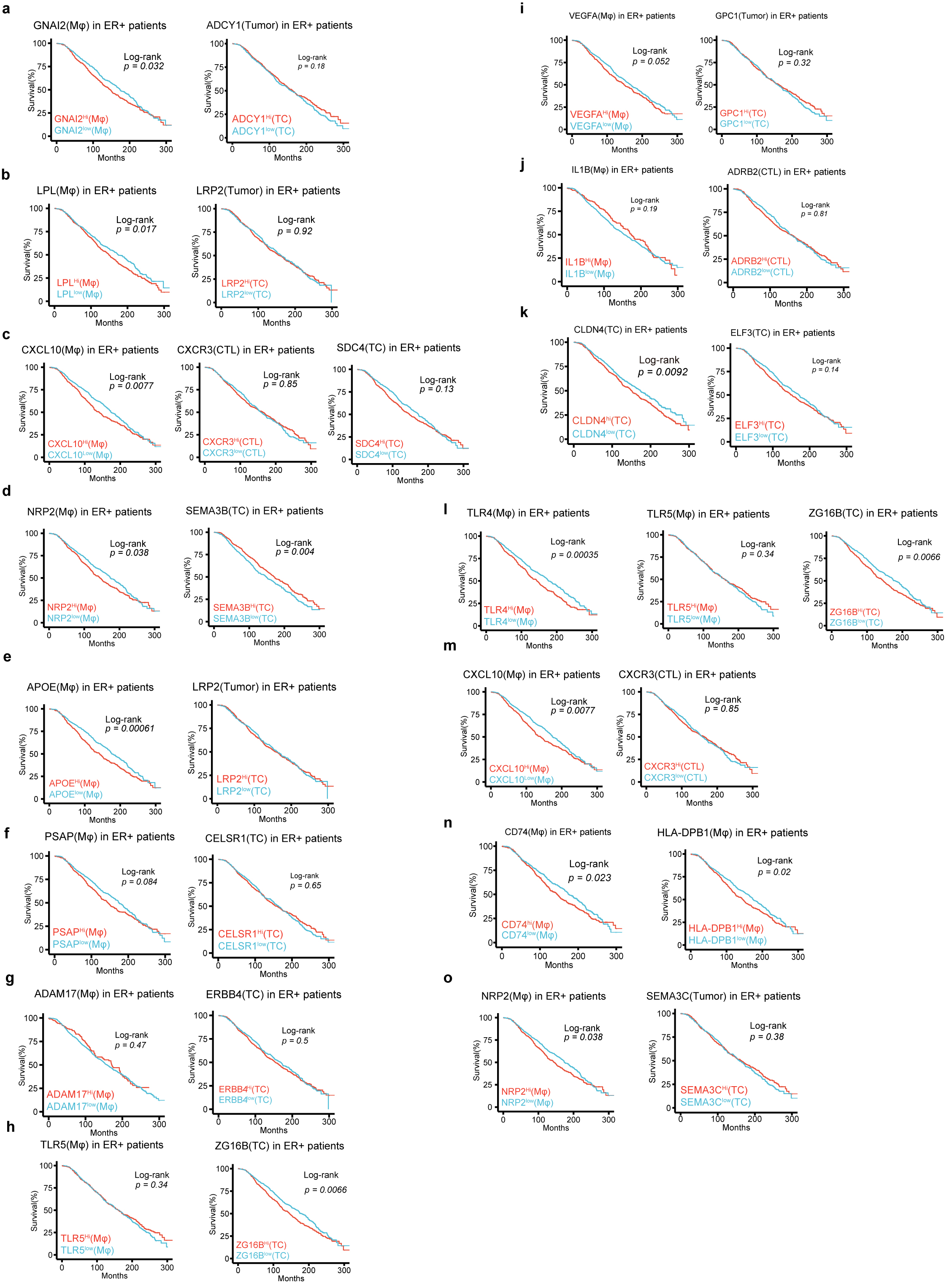


**Fig K. Survival analysis of genes in all *ESR1*-associated CCC signatures in ER+ METABRIC patient cohort. a-o** Kaplan-Meier analysis of overall survival outcomes in ER+ patients in METABRIC (n = 1196 patients in total) with various expression levels of gene in all significant *ESR1*-associated CCC signatures separately. All *P* values were computed by log-rank Test. Baseline curve represents the overall clinical outcome of all ER+ patients in METABRIC. CCC, cell-cell communication; TC, Tumor Cell; CTL, CD8+ T cell; Mφ, Macrophage.

Fig L. *ESR1*-associated CCC signatures identified using the breast dataset from Bassez et al..


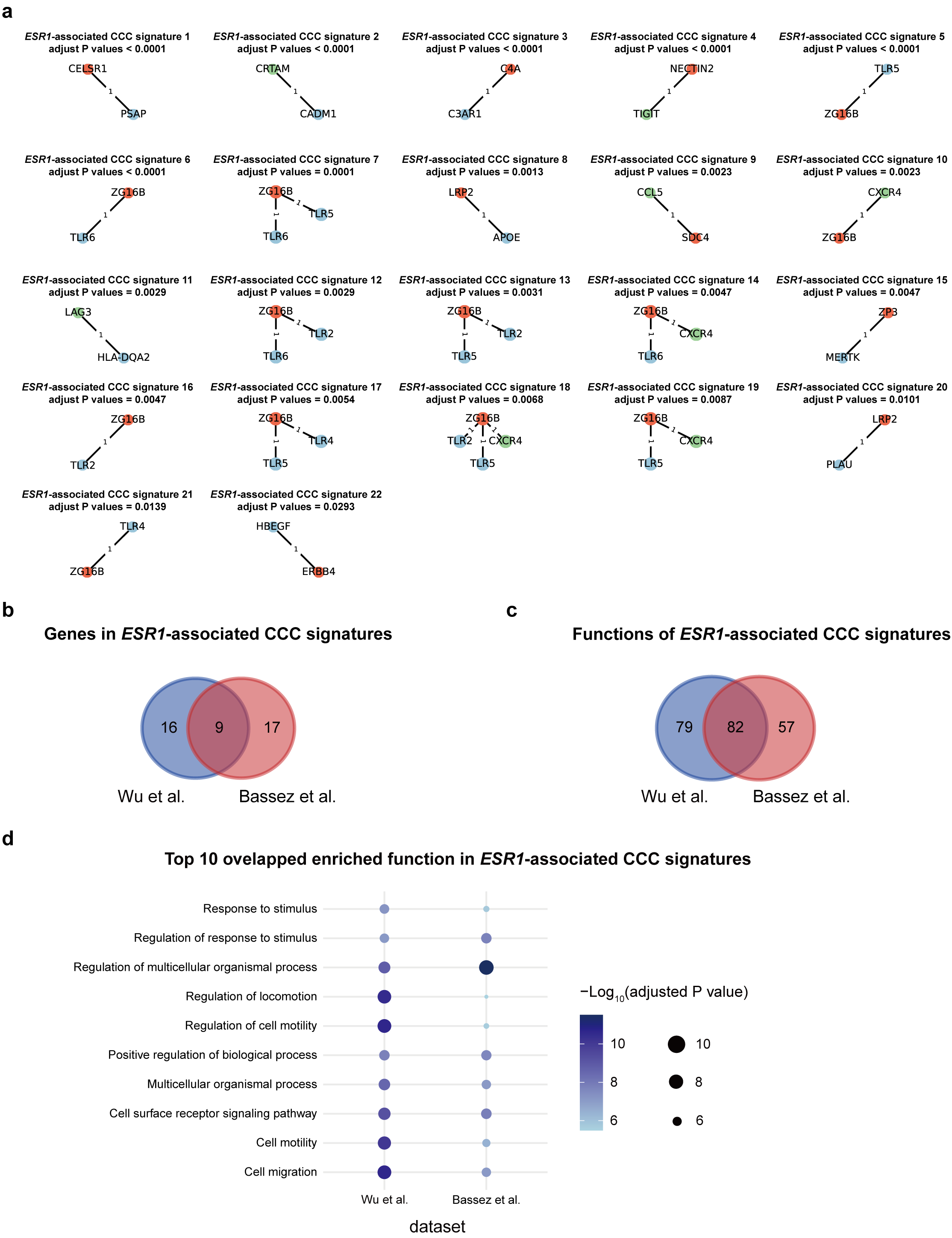


**Fig L. *ESR1*-associated CCC signatures identified using the dataset from the study by Bassez et al..** **a** Visualization of all *ESR1*-associated CCC signatures in this dataset. **b** Venn diagram shows the overlap among genes in *ESR1*-associated CCC signatures of the two breast cancer datasets. **c** Venn diagram shows the overlap among enriched functions of genes in *ESR1*-associated CCC signatures of the two breast cancer datasets. **d** The top 10 overlap enriched functions in the *ESR1*-associated CCC signatures of the two breast cancer datasets. *P* values of cancer driver-associated CCC signatures were computed using the Fisher’s exact test and adjusted using the method of Benjamini-Hochberg procedure. All CCC signatures were ranked according to their adjusted *P* values. Node labels are gene names and different colors are used to distinguish cell types with red for tumor cells, blue for macrophages, and green for CD8+ T cells. Edge labels represent different edge types with “0” for intracellular edges and “1” for intercellular edges.

Fig M. Survival analysis of all significant TNBC-associated CCC signatures in TNBC METABRIC patient cohort.


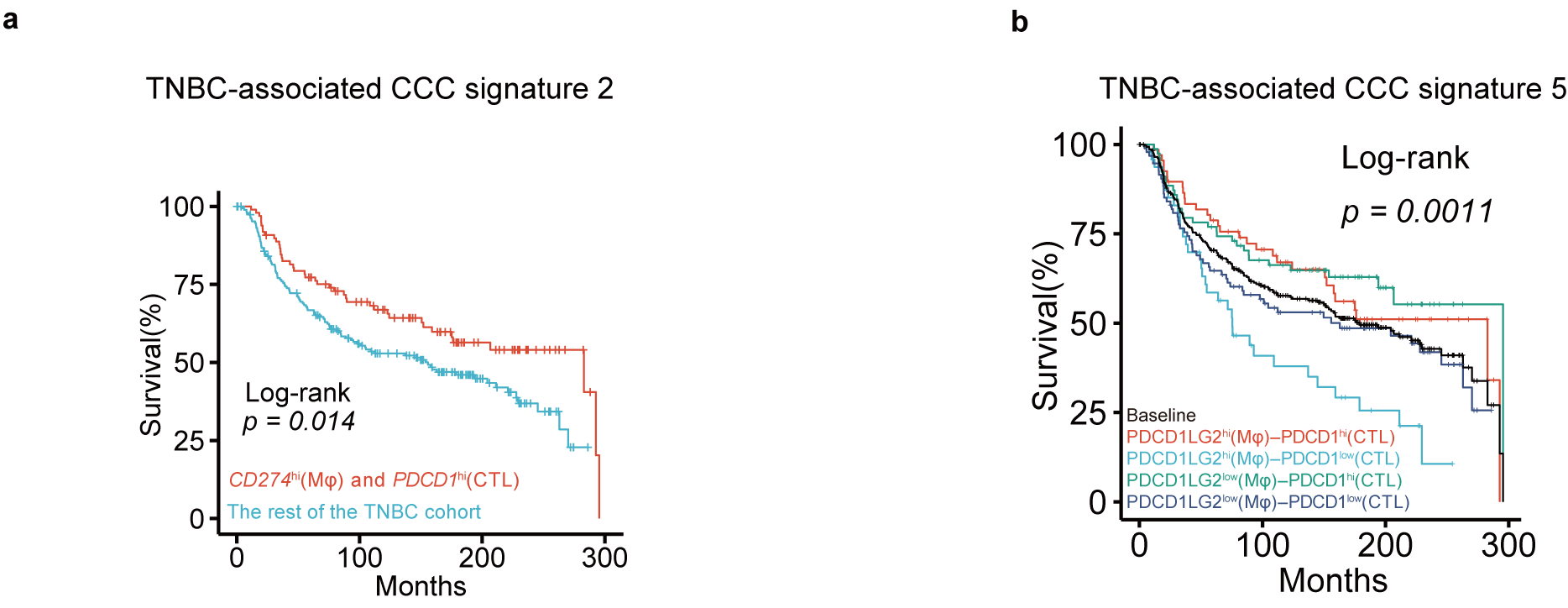


**Fig M. Survival analysis of all significant TNBC-associated CCC signatures in TNBC METABRIC patient cohort.** **a-b** Kaplan-Meier analysis of overall survival outcomes in patients in METABRIC (n = 290 patients in total) with various expression levels of all significant TNBC-associated CCC signatures. All *P* values were computed by Log-rank Test. Baseline curve represents the overall clinical outcome of all TNBC patients in METABRIC. Patients were stratified according to the expression of genes involved in TNBC-associated CCC signatures. For each gene in a given CCC signature, patients were categorized into highly-expressed and lowly-expressed groups based on the median expression value. All possible combinations of high/low states across the involved genes in the CCC signatures were considered, and each colored curve represents one such combinatorial expression pattern, as indicated in the legend. Survival differences among all stratified groups were evaluated using a multi-group log-rank test, and the reported *P* values correspond to the global comparison across all groups within each panel. CCC, cell-cell communication; TNBC, Triple Negative Breast Cancer; TC, Tumor Cell; CTL, CD8+ T cell; Mφ, Macrophage.

Fig N. Visualization of driver-associated CCC signatures in the neuroblastoma dataset.


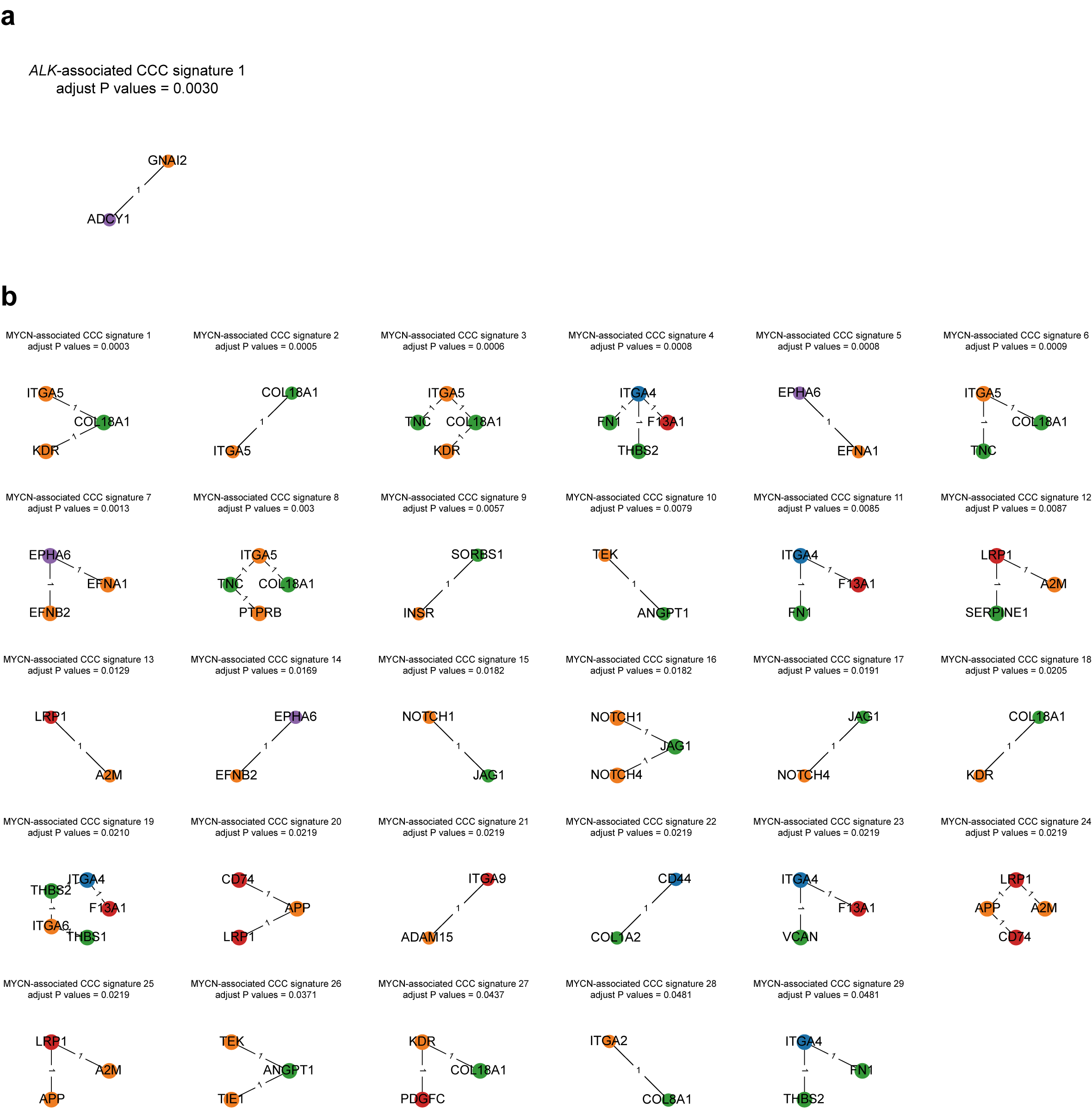


**Fig N. Visualization of driver-associated CCC signatures in the neuroblastoma dataset.** (**a-b**) CCC signatures significantly associated with *ALK* (**a**) and *MYCN* (**b**). *P* values of cancer driver-associated CCC signatures were computed using the Fisher’s exact test and adjusted using the method of Benjamini-Hochberg procedure. All CCC signatures were ranked according to their adjusted *P* values. Node labels are gene names and different colors are used to distinguish cell types with red for macrophage, blue for T cells, orange for endothelial cell, purple for tumor cell, and green for fibroblast. Edge labels represent different edge types with “0” for intracellular edges and “1” for intercellular edges.

Fig O. Visualization of CNV profile of the OSCC dataset.


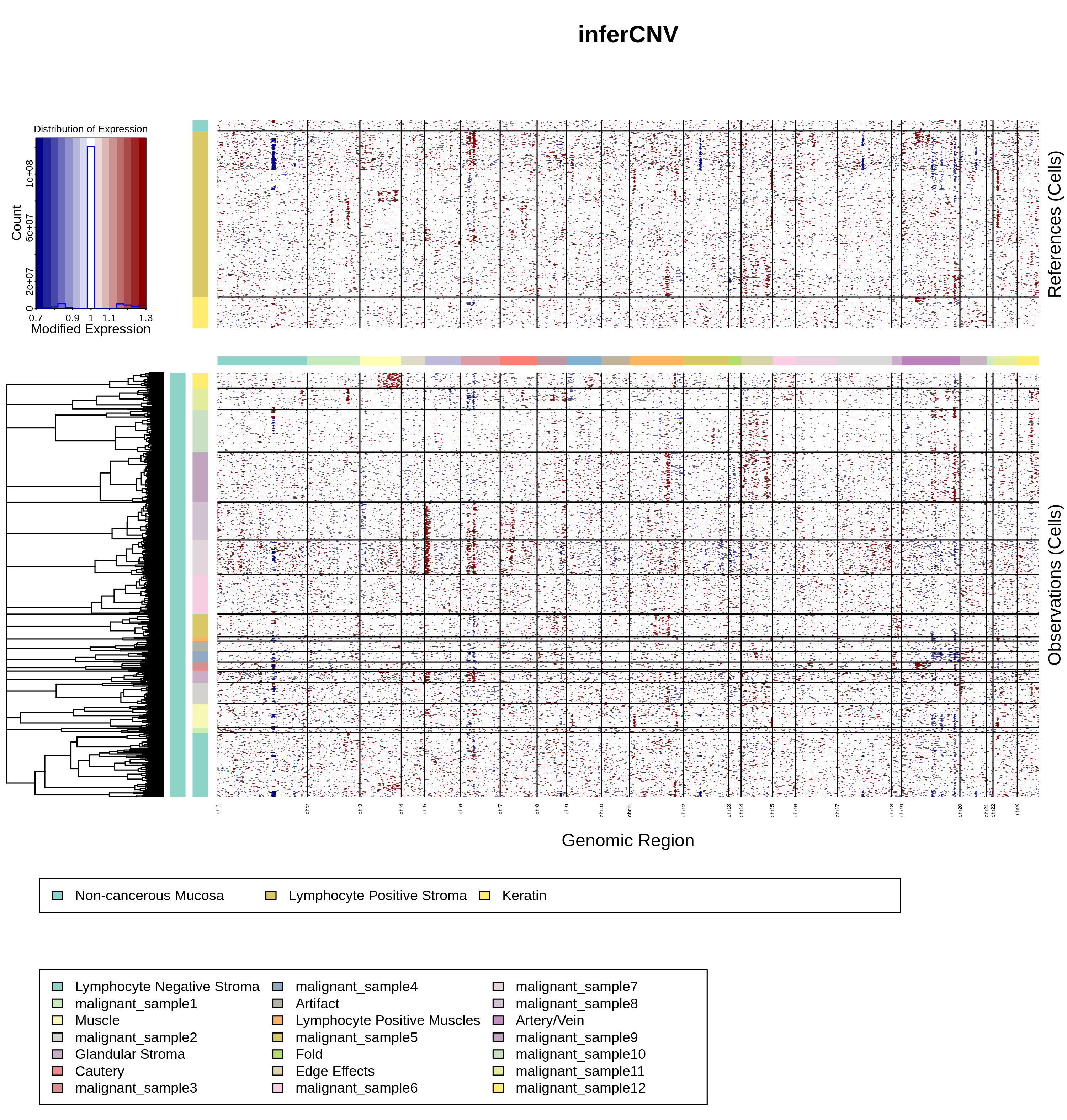


**Fig O. Visualization of CNV profile of the OSCC dataset. a** CNV profile in cancer versus references diploid spots assessed using SpatialInferCNV on the OSCC dataset.

Fig P. Visualization of driver-associated CCC signatures in the OSCC dataset.


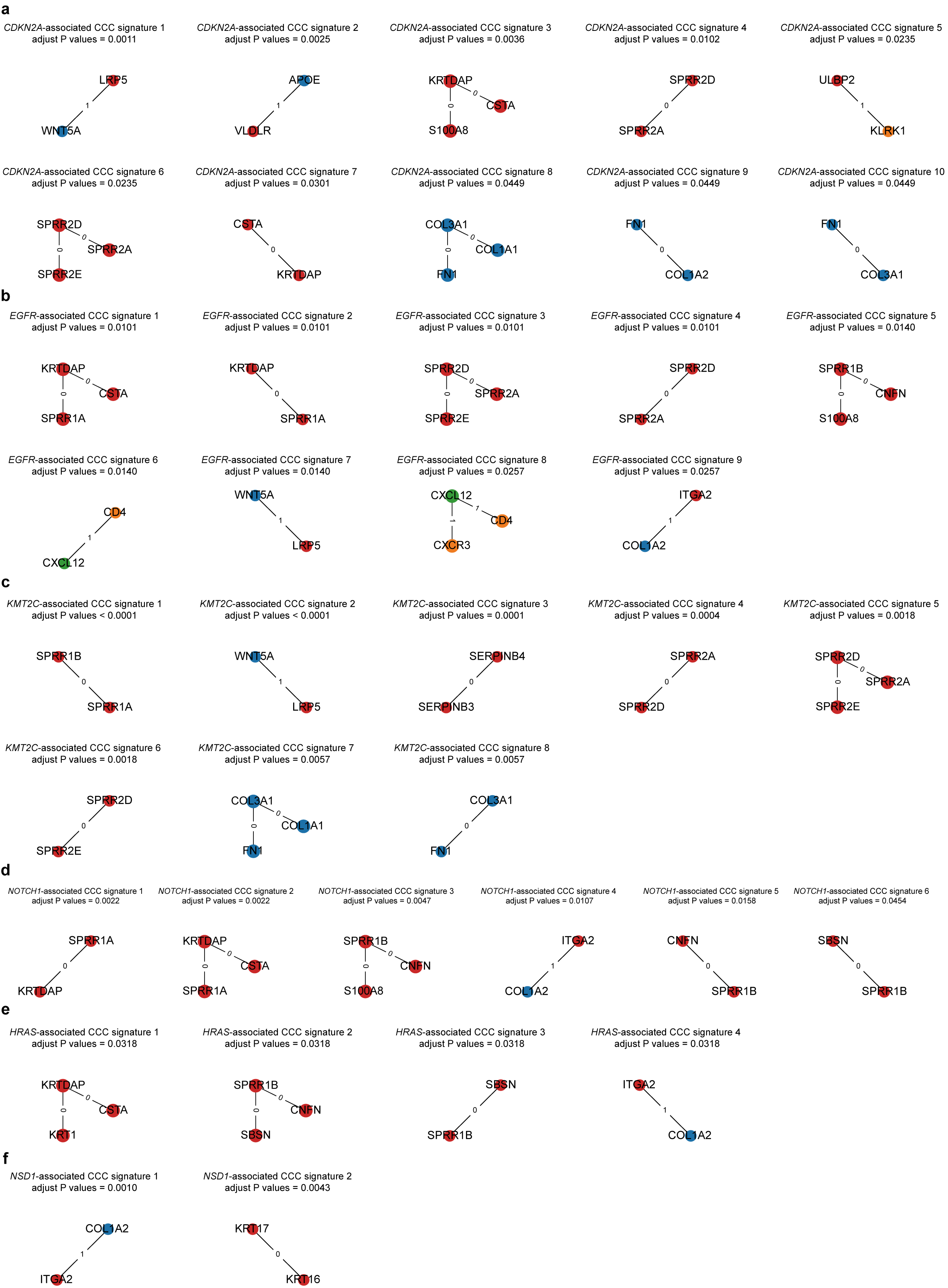


**Fig P. Visualization of driver-associated CCC signatures in the OSCC dataset.** (**a-f**) CCC signatures significantly associated with *CDKN2A* (**a**), *EGFR* (**b**), *KMT2C* (**c**), *NOTCH1* (**d**), *HRAS* (**e**), and *NSD1* (**f**). *P* values of cancer driver-associated CCC signatures were computed using the Fisher’s exact test and adjusted using the method of Benjamini-Hochberg procedure. All CCC signatures were ranked according to their adjusted *P* values. Node labels are gene names and different colors are used to distinguish cell types with red for squamous cell carcinoma (SCC), blue for lymphocyte-negative stroma (LNS), orange for lymphocyte-positive stroma (LPS), and green for muscle. Edge labels represent different edge types with “0” for intracellular edges and “1” for intercellular edges.

Fig Q. TNBC-associated CCC signatures identified using the breast dataset from Bassez et al..


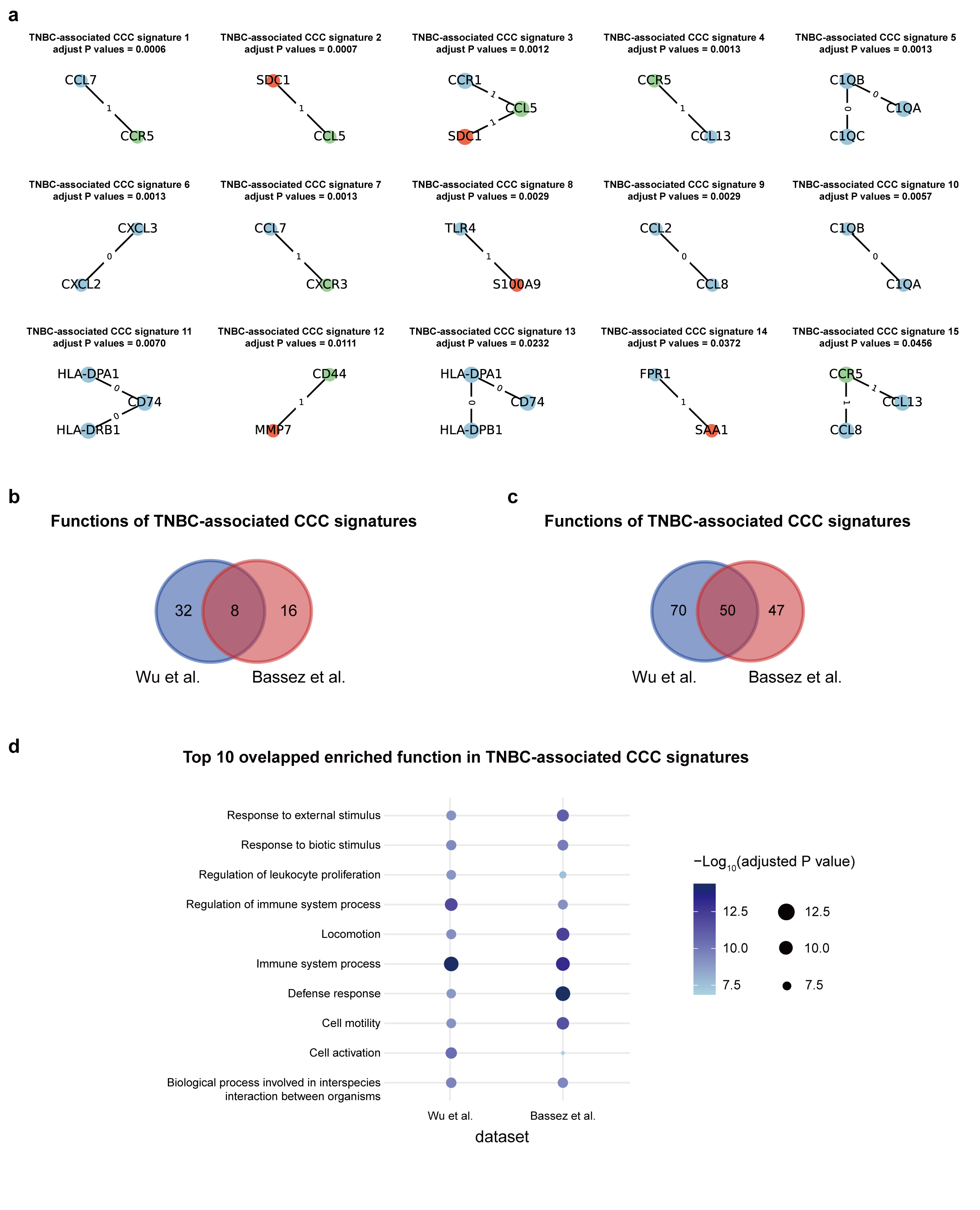


**Fig Q. TNBC-associated CCC signatures identified** **using the breast dataset from Bassez et al..** **a** Visualization of all TNBC-associated CCC signatures in this dataset. **b** Venn diagram shows the overlap among genes in TNBC-associated CCC signatures of the two breast cancer datasets. **c** Venn diagram shows the overlap among enriched functions of genes in TNBC-associated CCC signatures of the two breast cancer datasets. **d** The top 10 overlap enriched functions in the TNBC-associated CCC signatures of the two breast cancer datasets. *P* values of cancer driver-associated CCC signatures were computed using the Fisher’s exact test and adjusted using the method of Benjamini-Hochberg procedure. All CCC signatures were ranked according to their adjusted *P* values. Node labels are gene names and different colors are used to distinguish cell types with red for tumor cells, blue for macrophages, and green for CD8+ T cells. Edge labels represent different edge types with “0” for intracellular edges and “1” for intercellular edges. TNBC, Triple Negative Breast Cancer.

Fig R. Visualization of data integration and corresponding cancer driver-associated CCC signatures of breast cancer.


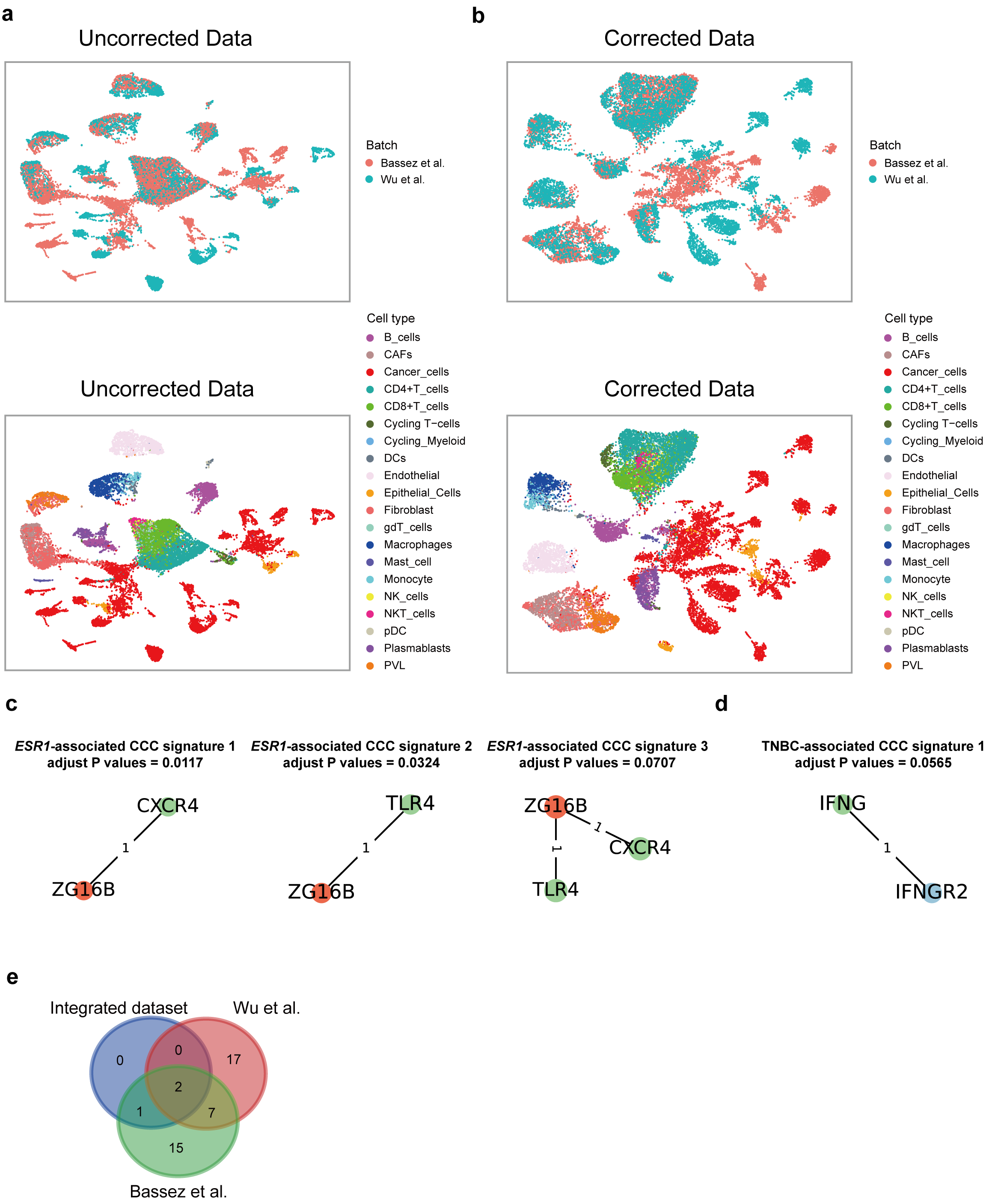


**Fig R. Visualization of data integration and corresponding cancer driver gene-associated CCC signatures of breast cancer. a-b** UMAP projection of the human breast cancer datasets before **(a)** and after **(b)** integration by Beaconet. **c** Visualization of all *ESR1*-associated CCC signatures identified using the integrated dataset. **d** Visualization of all TNBC-associated CCC signatures identified using the integrated dataset. **e** Venn diagram shows the overlap among genes in *ESR1*-associated CCC signatures identified using the three breast cancer datasets, respectively. *P* values of cancer driver-associated CCC signatures were computed using the Fisher’s exact test and adjusted using the method of Benjamini-Hochberg procedure. All CCC signatures were ranked according to their adjusted *P* values. Node labels are gene names and different colors are used to distinguish cell types with red for tumor cells, blue for macrophages, and green for CD8+ T cells. Edge labels represent different edge types with “0” for intracellular edges and “1” for intercellular edges. TNBC, Triple Negative Breast Cancer.

Fig S. Performance evaluation of Driver2Comm on the PDAC dataset.


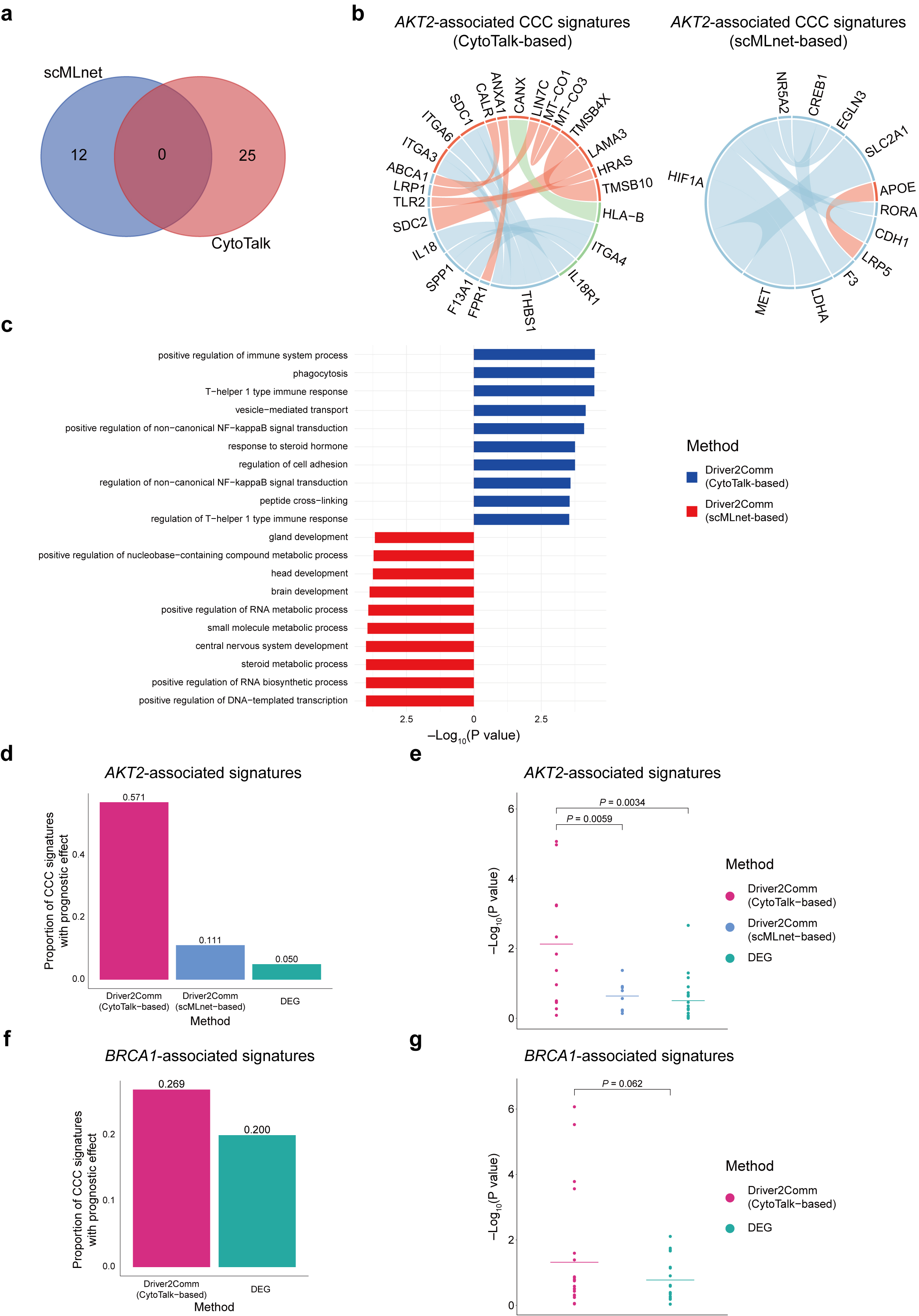


**Fig S.** **Performance comparison of CCC inference methods used in Driver2Comm on the PDAC dataset.** **a** Venn diagram showing the gene-level overlap of *AKT2*-associated CCC signatures identified by scMLnet-based and CytoTalk-based Driver2Comm. **b** Chord diagram visualization of *AKT2*-associated CCC signatures identified by CytoTalk-based Driver2Comm (left) and scMLnet-based Driver2Comm (right). Node represent genes, and colors indicate cell types: red for tumor cells, blue for macrophages, and green for CD8+ T cells. Edge colors indicate the sender cell type. Edge width reflected the association strength between cancer driver and corresponding driver gene-associated CCC signatures. It is inversely proportional to the adjusted *P* value. **c** Top uniquely enriched biological processes for CCC signatures identified by CytoTalk-based (blue) and scMLnet-based Driver2Comm (red). **d** Proportion of identified CCC signatures with significant prognostic effects across methods. **e** Performance evaluated by –log_10_ (*P* value) of log-rank test for survival associations of CCC signatures across methods or DEGs. **f** Proportion of identified *BRCA1*-associated CCC signatures with significant prognostic effects across methods. **g** Distribution of –log_10_ (*P* value) from log-rank tests for survival associations of *BRCA1*-associated CCC signatures across methods or *BRCA1*-associated DEGs. Each point corresponds to the performance on an identified CCC signatures or DEGs, with horizontal bars indicating the mean performance across all methods. *P* values for comparison were calculated using one-sided *t*-tests.

Fig T. Performance evaluation of Driver2Comm on the breast cancer dataset from Wu et al..


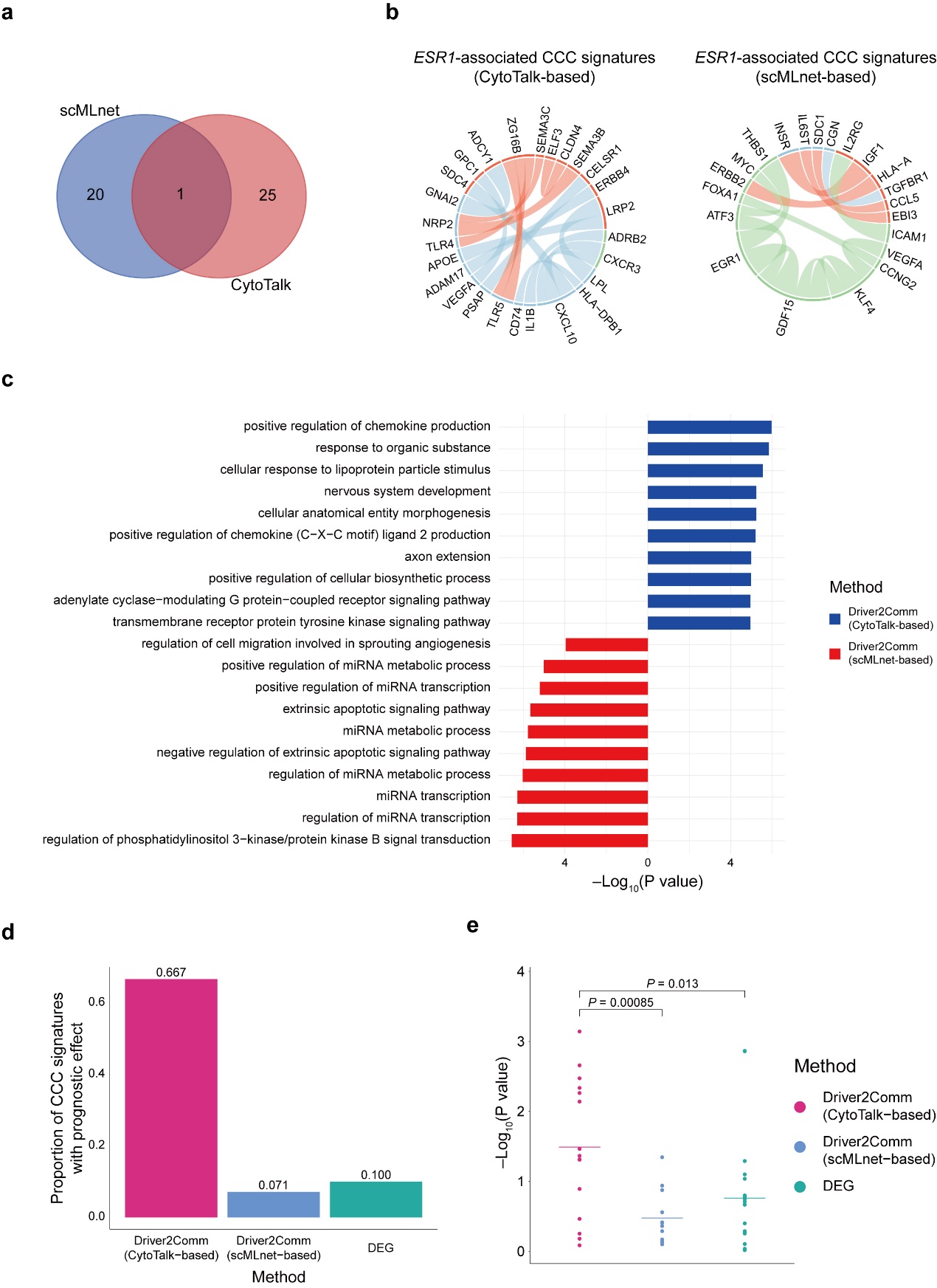


**Fig T.** **Performance evaluation of Driver2Comm on the breast cancer dataset from Wu et al..** **a** Venn diagram showing the gene-level overlap of *ESR1*-associated CCC signatures identified by scMLnet-based and CytoTalk-based Driver2Comm. **b** Chord diagram visualization of *ESR1*-associated CCC signatures identified by CytoTalk-based Driver2Comm (left) and scMLnet-based Driver2Comm (right). Node represent genes, and colors indicate cell types: red for tumor cells, blue for macrophages, and green for CD8+ T cells. Edge colors indicate the sender cell type. Edge width reflected the association strength between cancer driver and corresponding driver gene-associated CCC signatures. It is inversely proportional to the adjusted *P* value. **c** Top uniquely enriched biological processes for CCC signatures identified by CytoTalk-based (blue) and scMLnet-based Driver2Comm (red). **d** Proportion of identified CCC signatures with significant prognostic effects across methods. **e** Performance evaluated by – log_10_ (*P* value) of log-rank test for survival associations of CCC signatures across methods or DEGs. Each point corresponds to the performance on an identified CCC signatures or DEGs, with horizontal bars indicating the mean performance across all methods. *P* values for comparison were calculated using one-sided *t*-tests.

Fig U. Robustness assessment of Driver2Comm to cancer driver gene annotation errors.


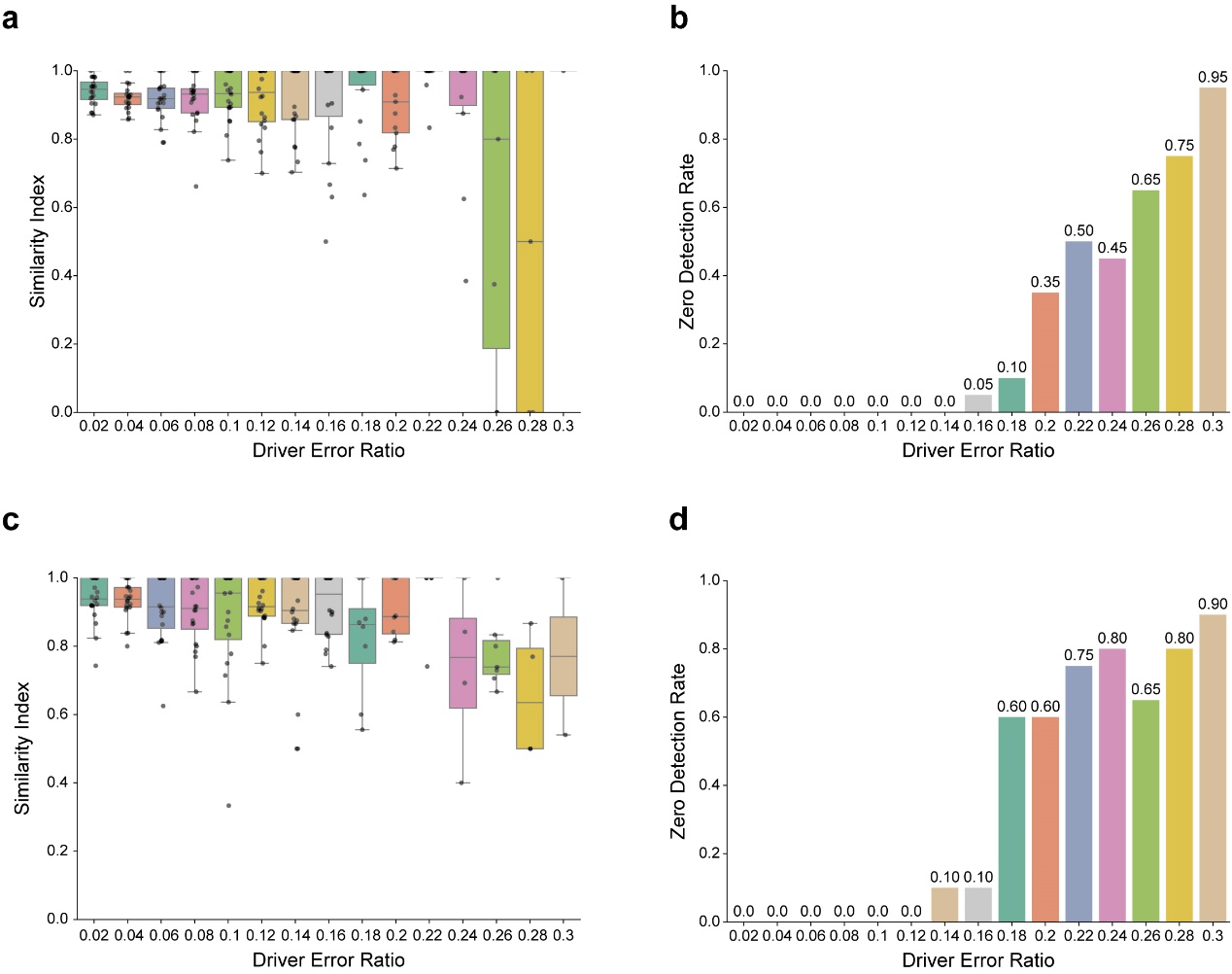


**Fig U.** **Robustness assessment of Driver2Comm to cancer driver gene annotation errors. (a-b)** Robustness analysis on the breast cancer dataset. Random driver annotation errors (2-30%) were introduced, and the identified driver gene-associated CCC signatures were compared with those obtained using the original driver labels. **(a)** Similarity index between CCC signatures identified under perturbed and unperturbed conditions across different driver error ratios. Each box represents results from repeated random perturbations. Each dot represents of a run with driver gene-associated CCC signature detected. **(b)** Zero detection rate, defined as the proportion of runs in which no driver gene-associated CCC signatures were identified, under increasing driver error ratios. **(c-d)** Robustness analysis on the neuroblastoma dataset. **(c)** Similarity index between CCC signatures identified under perturbed and unperturbed conditions across different driver error ratios. **(d)** Zero detection rate under increasing driver error ratios.

Table A. Identified driver information of patients in the PDAC dataset.

| Patients ID | Driver gene |
| --- | --- |
| U_1 | *KRAS,* |
| U_5 | *KRAS, MET, BRCA1* |
| U_6 | *KRAS,* *CDKN2A, MET, AKT2, BRCA1* |
| U_7 | *KRAS,* *CDKN2A* |
| U_8 | *KRAS, GATA6, BRCA1* |
| U_10 | *KRAS, MET, BRCA1* |
| U_12 | *KRAS,* *CDKN2A, GATA6, MET, BRCA1* |
| U_13 | *KRAS, GATA6, MET, AKT2* |
| U_15 | *KRAS,* *CDKN2A, GATA6, MET, AKT2, BRCA1* |
| U_18 | *KRAS,* *CDKN2A, GATA6, MET, AKT2* |

Table B. Driver information of patients in the breast cancer dataset from Wu et al..

| Patients ID | Subtype | Driver gene |
| --- | --- | --- |
| CID3921 | HER2+ | *ERBB2* |
| CID3948 | ER+ | *ESR1* |
| CID4067 | ER+ | *ESR1* |
| CID4290A | ER+ | *ESR1* |
| CID4463 | ER+ | *ESR1* |
| CID4471 | ER+ | *ESR1* |
| CID4495 | TNBC | Not identified |
| CID4515 | TNBC | Not identified |
| CID4530N | ER+ | *ESR1* |
| CID4535 | ER+ | *ESR1* |
| CID44971 | TNBC | Not identified |
| CID44991 | TNBC | Not identified |
| CID45171 | HER2+ | *ERBB2* |

Table C. Driver information of patients in the breast cancer dataset from Bassez et al..

| Patients ID | Subtype | Driver gene |
| --- | --- | --- |
| BIOKEY_1 | TNBC | Not identified |
| BIOKEY_2 | TNBC | Not identified |
| BIOKEY_3 | ER+ | *ESR1* |
| BIOKEY_4 | ER+ | *ESR1* |
| BIOKEY_5 | ER+ | *ESR1* |
| BIOKEY_6 | ER+ | *ESR1* |
| BIOKEY_7 | ER+ | *ESR1* |
| BIOKEY_8 | TNBC | Not identified |
| BIOKEY_9 | TNBC | Not identified |
| BIOKEY_10 | TNBC | Not identified |
| BIOKEY_11 | TNBC | Not identified |
| BIOKEY_12 | ER+ | *ESR1* |
| BIOKEY_13 | HER2+ | *ERBB2* |
| BIOKEY_14 | TNBC | Not identified |
| BIOKEY_15 | TNBC | Not identified |
| BIOKEY_16 | TNBC | Not identified |
| BIOKEY_17 | ER+ | *ESR1* |
| BIOKEY_18 | ER+ | *ESR1* |
| BIOKEY_19 | TNBC | Not identified |
| BIOKEY_21 | ER+ | *ESR1* |
| BIOKEY_24 | ER+ | *ESR1* |
| BIOKEY_25 | TNBC | Not identified |
| BIOKEY_26 | TNBC | Not identified |
| BIOKEY_27 | ER+ | *ESR1* |
| BIOKEY_28 | HER2+ | *ERBB2* |
| BIOKEY_29 | ER+ | *ESR1* |
| BIOKEY_30 | ER+ | *ESR1* |
| BIOKEY_31 | TNBC | Not identified |

Table D. Driver information of patients in the neuroblastoma dataset.

| Patients ID | Driver gene |
| --- | --- |
| CH_01 | Not identified |
| CH_02 | *ALK* |
| CH_03 | Not identified |
| CH_04 | *ALK* |
| CH_05 | *MYCN* |
| CH_06 | *ALK, MYCN* |
| CH_07 | Not identified |
| CH_08 | *MYCN* |
| CH_09 | Not identified |
| CH_10 | *ALK,* *MYCN* |
| CH_11 | Not identified |
| CH_12 | Not identified |
| CH_13 | *MYCN* |
| CH_14 | *MYCN* |
| CH_15 | Not identified |
| CH_16 | *MYCN* |
| CH_17 | Not identified |
| CH_18 | Not identified |
| CH_19 | *MYCN* |
| CH_20 | *MYCN* |
| CH_21 | Not identified |
| CH_22 | Not identified |
| CH_23 | *MYCN* |

Table E. Identified driver information of patients in the OSCC dataset.

| Patients ID | Driver gene |
| --- | --- |
| Sample_1 | Not identified |
| Sample_2 | *CCND1* |
| Sample_3 | Not identified |
| Sample_4 | *CCND1* |
| Sample_5 | *NOTCH1, HRAS, CCND1, KMT2C* |
| Sample_6 | *CDKN2A, FAT1, HRAS, CCND1* |
| Sample_7 | *FAT1, NOTCH1, HRAS, EGFR, NSD1* |
| Sample_8 | *KRAS, GATA6, MET, AKT2, EGFR* |
| Sample_9 | *CDKN2A,* *NOTCH1, CCND1, EGFR* |
| Sample_10 | *NOTCH1, HRAS, CCND1, EGFR* |
| Sample_11 | *CDKN2A, FAT1, CCND1, EGFR, KMT2C* |
| Sample_12 | *CDKN2A, NOTCH1, CCND1, EGFR, KMT2C* |

Supplementary Note

Note A, Additional supporting evidence for genes in *ESR1*-associated CCC signatures.

*GNAI2* can bias Macrophage Phenotype Determination. The M1 macrophage phenotype is promoted by *GNAI2* signaling, whereas an M2 phenotype is promoted by *GNAI2* signaling deficiency ^1^. A poor response to chemokines and chemoattractants was seen in *GNAI2*-deficient mice ^2^.

*LPL* encodes lipoprotein lipase, which is expressed in primary breast tumor tissues ^3^. By establishing a pro-tumorigenic cytokine secretion profile from tumor cells, Tobin A. J. et al. demonstrate that products of lipid hydrolysis from total lipoproteins by *LPL* may promote breast cancer growth and progression, which is independent of cell metabolic activity ^4^.

*CXCL10* is a protein-coding gene that stands for C-X-C motif chemokine ligand 10. *CXCL10* has been linked to breast cancer's promoted tumor cell proliferation, increased tumor lymphocytic infiltrates, and a bad prognosis for patients ^5–7^.

The proliferation, angiogenesis, and metastasis of cancer cells are all increased by autocrine *CXCL10*/*CXCR3* signaling. Previous studies have suggested that *CXCR3*-positive cancer cells may be more likely to metastasis as a result of autocrine signaling from the pre-metastatic niche both in vitro and in vivo ^7^. The *CXCL10*-*CXCR3* axis has been identified by Mulligan A M et al. as a possible therapeutic target for *BRCA1*-associated and basal breast tumors, which have a poor prognosis, a substantial lymphocytic infiltration, and are now in need of targeted therapy ^8^.

Through influencing cell proliferation, invasion, migration, metastasis formation, or angiogenesis, *SDC4* aids in the growth and spread of malignancies ^9–11^. Previous studies have linked *SDC4* to both a high histological grade and a negative status for the estrogen receptor, suggesting that it may be a predictor of a bad prognosis for breast cancer ^12^. Breast cancer cells in mice lacking *SDC4* exhibit a lower capacity for bone metastases ^13^.

*NRP2* (Neuropilin 2) encodes a member of the neuropilin family of receptor proteins. This protein may contribute to carcinogenesis, axon guidance, and cardiovascular development. According to Roy, et al., *NRP2* may serve as a molecular mediator between immune suppression and efferocytosis. *NRP2* deletion in TAM increased immune-stimulatory genes in the myeloid compartment while downregulating a number of immunosuppressive and tumor-promoting genes ^14^.

*SEMA3B* acts as a tumor suppressor in breast cancer. Through deactivating PI3K/Akt signaling, Sema3B inhibits tumor growth and triggers apoptosis in *NRP1*-positive breast cancer cells ^15,16^. The regulation of apoptosis in breast cancer cells is affected by the antagonistic interactions between Sema3B and *VEGF165* ^17^*.*

*APOE* (Apolipoprotein E) is a Protein Coding gene. T cells, macrophages, and natural killer T cells are only a few of the immune cells that produce ApoE ^18^ . According to a study conducted in vitro, ApoE may inhibit the growth of T cells. The innate antitumor response may be suppressed by elevated secreted ApoE proteins ^19^. Mice lacking in apoE can exacerbate the pro-inflammatory response brought on by lipopolysaccharide ^20^. According to Zhao B et al. ^21^, host ApoE deficiency could decrease tumor growth by enhancing cytotoxic T cell activity and preventing T cell exhaustion. Hence, blocking ApoE may be a successful cancer immunotherapy tactic.

*TLR5* knockdown increased tumor invasiveness in the TNBC ^22,23^.

*VEGF-A* is the key mediator of angiogenesis in cancer ^24^. Previous studies have shown that *VEGF-A* is highly up-regulated in breast cancer and expressed in TAM ^25^. The decidua's M2 polarization and macrophage recruitment may be aided by VEGF-A ^26^.

*CD74* is expressed by breast tumor cells as well as by several immune cell types. *CD74* acts as a macrophage inhibitory factor (MIF) to promote cell proliferation and migration ^27,28^.

*CLDN4* (Claudin-4) has been widely investigated in breast cancer ^29,30^. In vitro, claudin-4 inhibited breast cancer cells' ability to apoptosis while promoting their motility and proliferation ^31^. Furthermore, Claudin-4 positive was linked to a poor prognosis for breast cancer and a high tumor grade, indicating that Claudin-4 may be used as a biomarker for the early identification and diagnosis of breast cancer ^32–34^.

Note B, Performance comparison between scMLnet- and CytoTalk-based Driver2Comm.

To systematically evaluate how different CCC inference methods influence Driver2Comm, we applied scMLnet- and CytoTalk-based Driver2Comm to the PDAC dataset from Hwang et al.^36^ and the breast cancer dataset from Wu et al.^35^. We compared the biological characteristics and survival relevance of the resulting CCC signatures.

Biological differences between CCC signatures identified by the two methods were assessed from two complementary perspectives: (i) functional enrichment patterns and (ii) associations with patient survival. Survival relevance was evaluated using two metrics: (a) the proportion of identified CCC signatures or genes with significant prognostic effects, reflecting the recall of prognostically relevant signals; and (b) the distribution of –log_10_ (*P* values) from log-rank tests, measuring the overall strength of survival associations. Driver-associated differentially expressed genes (DEGs; top 20) were included as a baseline for comparison.

In the PDAC dataset, scMLnet-based Driver2Comm did not identify any *BRCA1*-associated CCC signatures; therefore, comparisons were performed only for *AKT2*-associated CCC signatures. Notably, CCC signatures identified by the two methods showed no gene-level overlap (Fig Sa). CytoTalk-based Driver2Comm predominantly detected **intercellular interactions**, whereas scMLnet-based Driver2Comm mainly identified **intracellular regulatory interactions** within macrophages (Fig Sb). Functional enrichment analysis further revealed distinct biological themes: as expected, CytoTalk-specific terms were enriched for **transmembrane signaling and immune-related processes**, such as vesicle-mediated transport, positive regulation of immune system process*,* T-helper 1 type immune response*,* and regulation of non-canonical NF-κB signaling. In contrast, scMLnet-specific terms were enriched for **intracellular regulatory pathways**, including RNA and DNA metabolic processes (Fig Sc). Consistent with these biological differences, CytoTalk-based Driver2Comm exhibited stronger survival relevance. In the *AKT2*-driven PDAC cohort, CytoTalk-based Driver2Comm identified a significantly higher proportion of prognostically relevant CCC signatures and exhibited significantly stronger overall survival associations than scMLnet-based Driver2Comm and DEGs (Figs Sd and Se). In the *BRCA1*-driven PDAC cohort, CytoTalk-based Driver2Comm showed superior average survival associations compared with DEGs, albeit without statistical significance (Figs Sf and Sg). scMLnet-based Driver2Comm was not included in this comparison because it did not identify any *BRCA1*-associated CCC signatures.

We next analyzed the breast cancer dataset. Consistent with the findings in the PDAC dataset, scMLnet- and CytoTalk-based Driver2Comm yielded markedly different *ESR1*-associated CCC signatures (Fig Ta). CytoTalk-based Driver2Comm primarily identified intercellular interactions between macrophages and tumor cells, whereas scMLnet-based Driver2Comm mainly captured intracellular regulatory pathways within CD8+ T cells (Fig Tb). Functional enrichment analysis further supported this divergence: CytoTalk-specific functional terms were enriched for processes related to transmembrane signaling and cellular responses to external stimuli, such as positive regulation of chemokine production, positive regulation of chemokine (C−X−C motif) ligand 2 production, and transmembrane receptor protein tyrosine kinase signaling pathway. In contrast, scMLnet-specific functional terms were enriched for pathways associated with gene expression regulation, RNA metabolism, and apoptosis, including regulation of miRNA metabolic process, positive regulation of miRNA transcription, and extrinsic apoptotic signaling pathway (Fig Tc). For survival relevance, CytoTalk-based Driver2Comm also achieved the best performance in the *ESR1*-driven cohort. Both the proportion of prognostically significant CCC signatures and the overall survival associations were significantly higher than those obtained using scMLnet-based Driver2Comm or DEGs (Figs Td and Te).

Together, these analyses demonstrate that CytoTalk-based Driver2Comm preferentially captures intercellular communication programs, whereas scMLnet-based Driver2Comm more frequently identifies intracellular regulatory pathways. Importantly, CytoTalk-based Driver2Comm consistently identifies CCC signatures with stronger and more robust prognostic relevance across datasets, highlighting the impact of CCC inference strategy on downstream biological interpretation and clinical association.

Note C, Robustness assessment of Driver2Comm.

To systematically evaluate the robustness of Driver2Comm to inaccuracies in cancer driver gene annotation using two independent datasets with known mutated drivers (i.e., a breast cancer dataset from Wu et al. ^35^ and a neuroblastoma dataset from Yu et al. ^40^).

Specifically, we simulated driver annotation errors by randomly introducing 2%–30% incorrect driver labels, and compared the resulting driver gene-associated CCC signatures with those obtained under the unperturbed (original) driver annotations. To quantitatively assess robustness under different levels of driver inaccuracy, we employed two complementary metrics.

1. Similarity index (SI)^41^ , which measures the consistency between CCC signatures identified under perturbed and unperturbed conditions:

$$SI=\frac{\left| A\cap B \right|}{min(\left| A \right|,\left| B \right|)}$$

where A and B denote the sets of genes in driver gene-associated CCC signatures identified with and without driver perturbation, respectively.

1. Zero detection rate, which quantifies the proportion of runs in which no driver gene-associated CCC signatures were identified. Importantly, this metric reflects false-positive control: when driver annotations are excessively corrupted, and the true association between driver genes and CCC signatures is disrupted, we expect Driver2Comm not to report spurious associations, but instead to return no result.

We first evaluated robustness to driver label inaccuracies using the breast cancer dataset. The identified CCC signatures remained highly consistent until the driver error rate exceeded 26% (Fig Ua). Meanwhile, when the error rate exceeded 16%, Driver2Comm progressively began to return no driver gene-associated CCC signatures. Notably, at a 30% error rate, the Zero detection rate reached 95%, indicating strong suppression of false-positive discoveries (Fig Ub). In the neuroblastoma dataset, CCC signatures remained robust across a wide range of perturbation levels (Fig Uc). Similarly, once the error rate exceeded 16%, the Zero detection rate increased steadily with increasing perturbation (Fig Ud), again demonstrating effective false-positive control.

In summary, these results demonstrate that Driver2Comm is highly robust to moderate levels of driver gene annotation errors, maintaining consistent CCC identification even under substantial perturbation. More importantly, Driver2Comm exhibits excellent false-positive control: when driver annotations become unreliable, the method tends to return no identified driver gene-associated CCC signatures instead of producing misleading results.

Note D, Rule of patient selection in this study.

In this study, we selected patient samples for analysis based on three specific criteria:

(1) The tumor samples must be derived from patients with primary, untreated breast cancer.

(2) Each tumor sample must contain a minimum of 10 macrophages, CD8+ T cells, and cancer cells.

(3) Each tumor sample must have expression of at least 1000 genes across more than three cells in macrophages, CD8+ T cells, and cancer cells.

Previous studies have demonstrated the CCC patterns will alter during metastasis and treatments, thus we set criterion 1 to minimize the influence of our results by these confounding factors. Criterion 2 is designed to ensure sufficient representation of key cell types for robust CCC patterns identification and criterion 3 is designed to ensure high data quality for subsequent analyses.

Hence, in dataset from Hwang et al.^36^, patient U2, U3, U4, U9, U11, U14, U16, U17 were excluded for not satisfying criteria (2). In dataset from Wu et al., patient CID4523, CID4513, CID3963, CID4066, CID4398 were excluded for not satisfying criteria (1), CID3586, CID3838, CID3946, CID4040, CID44041 were excluded for not satisfying criteria (2), CID3941, CID4461, CID4465 were excluded for not satisfying criteria (3). In dataset from Bassez et al.^37^, patient BIOKEY_20, BIOKEY_22, BIOKEY_23, were excluded for not satisfying criteria (2). For the neuroblastoma and OSCC datasets, all samples were retained for downstream analyses.

While this study focuses on primary, untreated tumor samples to reduce confounding factors, Driver2Comm is flexible to be applied to various experimental settings. For example, users can apply Driver2Comm to compare untreated and treated tumor samples to investigate how driver gene-associated CCC signatures change after treatment, providing insights into treatment-induced alterations in tumor-immune interactions. Similarly, Driver2Comm can be used to compare metastatic and primary tumor samples to explore CCC changes associated with metastasis, which could aid in designing more personalized therapeutic strategies.

Note E, Parameter selection in this study.

The principle for selecting the minimum support (min sup) was to control the number of frequent subgraphs to approximately 200. For association testing, we set the threshold to 0.05, except for the PDAC dataset. The threshold in PDAC dataset was set to 0.1 because smaller thresholds resulted in fewer identified CCC signatures, which could hinder subsequent analyses. The specific parameters are as follows:

For CytoTalk-based Driver2Comm:

1. PDAC dataset (Hwang et al.): Min sup = 8; Association testing threshold = 0.1.
2. Breast Cancer dataset (Wu et al.): Min sup = 14; Association testing threshold = 0.05.
3. Breast Cancer dataset (Bassez et al.):
   1. For the whole dataset: Min sup = 20; Association testing threshold = 0.05.
   2. For the NE group: Min sup = 10; Association testing threshold = 0.05.
   3. For the E group: Min sup = 5; Association testing threshold = 0.05."
4. OSCC dataset (Arora et al.): Min sup = 8; Association testing threshold = 0.05.
5. Neuroblastoma dataset (Yu et al.): Min sup = 20; Association testing threshold = 0.05.

For scMLnet-based Driver2Comm:

1. PDAC dataset (Hwang et al.): Min sup = 20; Association testing threshold = 0.1.
2. Breast Cancer dataset (Wu et al.): Min sup = 20; Association testing threshold = 0.05.

Note F, Deconvolution of bulk RNA profile data.

The cell type annotation derived from the Wu et al.^35^ includes *Cancer Epithelial cells, Normal Epithelial cells, Endothelial cells, cancer-associated fibroblast (CAF), perivascular-like (PVL), Plasmablasts, Macrophages, Monocytes, Cycling Myeloids, Dendritic cells(DCs),CD4+ T cells,CD8+ T cells, Cycling T cells, NK cells, NKT cells, B cells.*

Note G, Candidate cancer driver of different cancer type.

PDAC: Candidate cancer driver genes collected from Connor et al**.**^38^**,** including *KRAS*, TP53, *CDKN2A*, SMAD4, *GATA6*, *MET*, *NOTCH1*, *ERBB2*, *AKT2*, *MYC*, *BRCA1*, *ARID1A*, *PTEN, PBRM1, SMARCA4*

OSCC: Candidate cancer driver genes collected from Tan et al.^39^, including *TP53*, *CDKN2A*, *FAT1*, *NOTCH1*, *CASP8*, *HRAS*, *EGFR*, *KMT2C*, *NSD1*. *TP53* and *CASP8* did not pass the SpatialInferCNV gene filter, so we performed Driver2Comm testing on the remaining eight genes.

$$SI=\frac{\left| A\cap B \right|}{min(\left| A \right|,\left| B \right|)}$$

Supplemental References

1. Vural, A. *et al.* Gαi2 Signaling Regulates Inflammasome Priming and Cytokine Production by Biasing Macrophage Phenotype Determination. *The Journal of Immunology* **202**, 1510–1520 (2019).

2. Kehrl, J. H. The impact of RGS and other G-protein regulatory proteins on Gαi-mediated signaling in immunity. *Biochemical Pharmacology* **114**, 40–52 (2016).

3. Kuemmerle, N. B. *et al.* Lipoprotein Lipase Links Dietary Fat to Solid Tumor Cell Proliferation. *Molecular Cancer Therapeutics* **10**, 427–436 (2011).

4. Tobin, A. J., Noel, N. P., Christian, S. L. & Brown, R. J. Lipoprotein lipase hydrolysis products induce pro-inflammatory cytokine expression in triple-negative breast cancer cells. *BMC Res Notes* **14**, 315 (2021).

5. Szeto, E. & Kim, S. Abstract 3453: CXCL10 contributes to aggressive disease progression in ING4-deficient breast cancer. *Cancer Research* **79**, 3453 (2019).

6. Kim, M., Choi, H. Y., Woo, J. W., Chung, Y. R. & Park, S. Y. Role of CXCL10 in the progression of in situ to invasive carcinoma of the breast. *Sci Rep* **11**, 18007 (2021).

7. Tokunaga, R. *et al.* CXCL9, CXCL10, CXCL11/CXCR3 axis for immune activation – A target for novel cancer therapy. *Cancer Treatment Reviews* **63**, 40–47 (2018).

8. Mulligan, A. M. *et al.* Tumoral Lymphocytic Infiltration and Expression of the Chemokine CXCL10 in Breast Cancers from the Ontario Familial Breast Cancer Registry. *Clinical Cancer Research* **19**, 336–346 (2013).

9. Yip, G. W., Smollich, M. & Götte, M. Therapeutic value of glycosaminoglycans in cancer. *Molecular Cancer Therapeutics* **5**, 2139–2148 (2006).

10. Espinoza-Sánchez, N. A. & Götte, M. Role of cell surface proteoglycans in cancer immunotherapy. *Seminars in Cancer Biology* **62**, 48–67 (2020).

11. Hassan, N., Greve, B., Espinoza-Sánchez, N. A. & Götte, M. Cell-surface heparan sulfate proteoglycans as multifunctional integrators of signaling in cancer. *Cellular Signalling* **77**, 109822 (2021).

12. Baba, F. *et al.* Syndecan-1 and syndecan-4 are overexpressed in an estrogen receptor-negative, highly proliferative breast carcinoma subtype. *Breast Cancer Res Treat* **98**, 91–98 (2006).

13. Leblanc, R., Sahay, D., Houssin, A., Machuca-Gayet, I. & Peyruchaud, O. Autotaxin-β interaction with the cell surface via syndecan-4 impacts on cancer cell proliferation and metastasis. *Oncotarget* **9**, 33170–33185 (2018).

14. Roy, S. *et al.* Macrophage-Derived Neuropilin-2 Exhibits Novel Tumor-Promoting Functions. *Cancer Research* **78**, 5600–5617 (2018).

15. Butti, R., Kumar, T. V., Nimma, R. & Kundu, G. C. Impact of semaphorin expression on prognostic characteristics in breast cancer. *Breast Cancer: Targets and Therapy* https://www.tandfonline.com/doi/abs/10.2147/BCTT.S135753 (2018).

16. Castro-Rivera, E., Ran, S., Thorpe, P. & Minna, J. D. Semaphorin 3B (SEMA3B) induces apoptosis in lung and breast cancer, whereas VEGF_165_ antagonizes this effect. *Proc. Natl. Acad. Sci. U.S.A.* **101**, 11432–11437 (2004).

17. Castro-Rivera, E., Ran, S., Brekken, R. A. & Minna, J. D. Semaphorin 3B Inhibits the Phosphatidylinositol 3-Kinase/Akt Pathway through Neuropilin-1 in Lung and Breast Cancer Cells. *Cancer Research* **68**, 8295–8303 (2008).

18. Zhang, H.-L., Wu, J. & Zhu, J. The Immune-Modulatory Role of Apolipoprotein E with Emphasis on Multiple Sclerosis and Experimental Autoimmune Encephalomyelitis. *Journal of Immunology Research* **2010**, 186813 (2010).

19. Tavazoie, M. F. *et al.* LXR/ApoE Activation Restricts Innate Immune Suppression in Cancer. *Cell* **172**, 825-840.e18 (2018).

20. Oosten, M. V. *et al.* Apolipoprotein E Protects Against Bacterial Lipopolysaccharide-induced Lethality: A NEW THERAPEUTIC APPROACH TO TREAT GRAM-NEGATIVE SEPSIS *. *Journal of Biological Chemistry* **276**, 8820–8824 (2001).

21. Zhao, B., Wang, W., Chen, Z. & Jin, S. Apolipoprotein E deficiency enhances the anti-tumor immunity via repressing T cell exhaustion. *All Life* **14**, 1054–1062 (2021).

22. Shi, D. & Shi, H. Toll-like receptor 5 in triple-negative breast cancer: a novel reporter for tumor progression. *Journal of Nuclear Medicine* **62**, 65–65 (2021).

23. Shi, D. *et al.* TLR5: A prognostic and monitoring indicator for triple-negative breast cancer. *Cell Death Dis* **10**, 1–11 (2019).

24. Carmeliet, P. VEGF as a Key Mediator of Angiogenesis in Cancer. *Oncology* **69**, 4–10 (2005).

25. Liu, Y. *et al.* The association between vascular endothelial growth factor expression in invasive breast cancer and survival varies with intrinsic subtypes and use of adjuvant systemic therapy: results from the Nurses’ Health Study. *Breast Cancer Res Treat* **129**, 175–184 (2011).

26. Wheeler, K. C. *et al.* VEGF may contribute to macrophage recruitment and M2 polarization in the decidua. *PLOS ONE* **13**, e0191040 (2018).

27. Bucala, R. & Shachar, I. The Integral Role of CD74 in Antigen Presentation, MIF Signal Transduction, and B Cell Survival and Homeostasis. *MRMC* **14**, 1132–1138 (2015).

28. Leng, L. *et al.* MIF Signal Transduction Initiated by Binding to CD74. *Journal of Experimental Medicine* **197**, 1467–1476 (2003).

29. Morin, P. J. Claudin Proteins in Human Cancer: Promising New Targets for Diagnosis and Therapy. *Cancer Research* **65**, 9603–9606 (2005).

30. Soini, Y. Expression of claudins 1, 2, 3, 4, 5 and 7 in various types of tumours. *Histopathology* **46**, 551–560 (2005).

31. Ma, X. *et al.* Claudin-4 controls the proliferation, apoptosis, migration and in vivo growth of MCF-7 breast cancer cells. *Oncology Reports* **34**, 681–690 (2015).

32. Lanigan, F. *et al.* Increased claudin-4 expression is associated with poor prognosis and high tumour grade in breast cancer. *International Journal of Cancer* **124**, 2088–2097 (2009).

33. Kolokytha, P. *et al.* Claudin-3 and Claudin-4: Distinct Prognostic Significance in Triple-Negative and Luminal Breast Cancer. *Applied Immunohistochemistry & Molecular Morphology* **22**, 125–131 (2014).

34. Szasz, A. M. *et al.* Identification of a claudin-4 and E-cadherin score to predict prognosis in breast cancer. *Cancer Science* **102**, 2248–2254 (2011).

35. Wu, S. Z. *et al.* A single-cell and spatially resolved atlas of human breast cancers. *Nat Genet* **53**, 1334–1347 (2021).

36. Hwang, W. L. *et al.* Single-nucleus and spatial transcriptome profiling of pancreatic cancer identifies multicellular dynamics associated with neoadjuvant treatment. *Nat Genet* **54**, 1178–1191 (2022).

37. Bassez, A. A single-cell map of intratumoral changes during anti-PD1 treatment of patients with breast cancer. *Nature Medicine* **27**, (2021).

38. Connor, A. A. & Gallinger, S. Pancreatic cancer evolution and heterogeneity: integrating omics and clinical data. *Nat Rev Cancer* **22**, 131–142 (2022).

39. Tan, Y. *et al.* Oral squamous cell carcinomas: state of the field and emerging directions. *Int J Oral Sci* **15**, 44 (2023).

40. Yu, W. *et al.* Longitudinal single-cell multiomic atlas of high-risk neuroblastoma reveals chemotherapy-induced tumor microenvironment rewiring. *Nat Genet* **57**, 1142–1154 (2025).

41. Luo, J., Deng, M., Zhang, X. & Sun, X. ESICCC as a systematic computational framework for evaluation, selection, and integration of cell-cell communication inference methods. *Genome Res.* **33**, 1788–1805 (2023).
