# Supplementary material for: Bridging cancer cell-intrinsic driver genes and -extrinsic cell-cell communication with Driver2Comm
Source: PLoS Comput Biol. 2026 Feb 17;22(2):e1013973. doi: 10.1371/journal.pcbi.1013973 (PMC12928580; doi:10.1371/journal.pcbi.1013973)
Supplement: Attachment — Submitted filename: Response_to_reviewer_comments_20251222.pdf [file pcbi.1013973.s003.pdf]

Response to reviewers

We would like to sincerely thank the editor and the reviewers for their constructive comments and insightful suggestions, which have greatly helped us improve both the clarity and quality of our manuscript. In the following, reviewers' comments remain in **black**, our point-by-point answers appear in **blue**. Text and figures we have added or altered in the main manuscript are also highlighted in **blue**.

Reviewer #1:

This manuscript presents Driver2Comm, a novel computational framework that systematically bridges cancer cell-intrinsic driver genes and extrinsic cell-cell communication (CCC) events in the tumor microenvironment (TME). The study addresses a critical gap in cancer biology by integrating single-cell transcriptomics, functional pathway analysis, and clinical validation to uncover intrinsic-extrinsic (IE) pathways with remarkable biological and translational relevance.

Driver2Comm is the first tool to explicitly link driver genes to CCC signatures via de novo reconstructed multi-cell-type communication (MCTC) networks, leveraging CytoTalk for intracellular/intercellular signaling and GWAS-inspired statistical association testing. This approach elegantly overcomes limitations of existing tools. The application to three distinct cancer types (PDAC, ER+/HER2+ breast cancer, TNBC) demonstrates broad utility. Key findings—such as BRCA1-mediated immunosuppression in PDAC, ER1-associated CCC signatures predicting anti-PD1 response, and the paradoxical prognostic role of CD274-PDCD1 in TNBC—are robustly supported by survival analysis, immune scoring, and independent cohort validation.

Minor suggestions:

R1C1

1. Please clarify the rationale for selecting macrophages and CD8+ T cells as focal immune populations, given the potential contribution of other stromal cells (e.g., fibroblasts, dendritic cells).

We thank the reviewer for raising this important point. In the original manuscript, we selected macrophages and CD8+ T cells as representative immune cell types to construct the MCTC network for the following three reasons:

(1) **Key players in immune responses.** Macrophages and CD8+ T cells are central components of innate and adaptive immunity, respectively. Their interactions with cancer cells can either suppress or promote tumor progression, reflecting the coordinated interplay between innate and adaptive immunity. Focusing on these two cell types therefore provides a representative overview of immune responses

in cancer [1–3].

(2) Closely interacting cell types. The crosstalk among macrophages, cancer cells, and CD8+ T cells has been extensively studied and is considered crucial for coordinating anti-tumor immunity [4–8]. For example, macrophages can present antigens and secrete cytokines that modulate CD8+ T cell activation and effector function [7]. Conversely, CD8+ T cells can affect macrophage polarization and function by releasing cytokines [9,10].

(3) Relevance to cancer prognosis and therapy. Both macrophages and CD8+ T cells play pivotal roles in cancer immunotherapy and patient prognosis. Previous studies indicated that targeting these cell types has shown promising results in cancer therapy [11–13]. Identifying CCC signatures involving macrophages and CD8+ T cells may thus facilitate the discovery of novel prognostic biomarkers and immunotherapy targets.

We also acknowledge that clinically relevant CCC signatures involving other cell types in the TME could have been overlooked. To address this concern, in the revised manuscript, we expanded our analysis to a neuroblastoma dataset **comprising eight major cell types** and annotation of *MYCN* status. Using Driver2Comm, we identified 29 *MYCN*-associated CCC signatures that displayed functional modularity specific to distinct cell-type pairs. Notably,

- **Fibroblast-to-endothelial cell** communication emerged as the dominant *MYCN*-associated CCC signatures (Response Fig. 1a). These signatures were enriched for angiogenesis-related processes (Response Fig. 1b), aligned with the previous findings linking *MYCN* amplification to increased vascularity in neuroblastoma [14–16].
- **Endothelial cell-to-macrophage** interactions revealed distinct immune regulatory programs, including immune cell migration, locomotion, and immune system processes (Response Fig. 1b), consistent with the known role of endothelial cells in myeloid cell recruitment and positioning within the TME [17,18].
- Only limited **T cell-involving** *MYCN*-associated CCC signatures were observed (Response Fig. 1a), which may reflect immunologically "cold" status in the TME of *MYCN* amplification neuroblastoma[19–21].
- **Fibroblast-to-endothelial cell** interactions were enriched for the ephrin receptor signaling pathway (Response Fig. 1b), which was well known for its roles in neural and vascular development. Prior studies have linked ephrin signaling components with *MYCN* amplification and prognosis in neuroblastoma [22,23].

Taken together, these results demonstrate that Driver2Comm can naturally capture higher-order CCC architectures involving multiple stromal and immune cell types beyond macrophages and CD8+ T cells.

Full details of neuroblastoma analysis are provided in the Results section under the heading: **"*MYCN*-associated CCC signatures exhibit cell-type-pair-specific functional modularity"**.

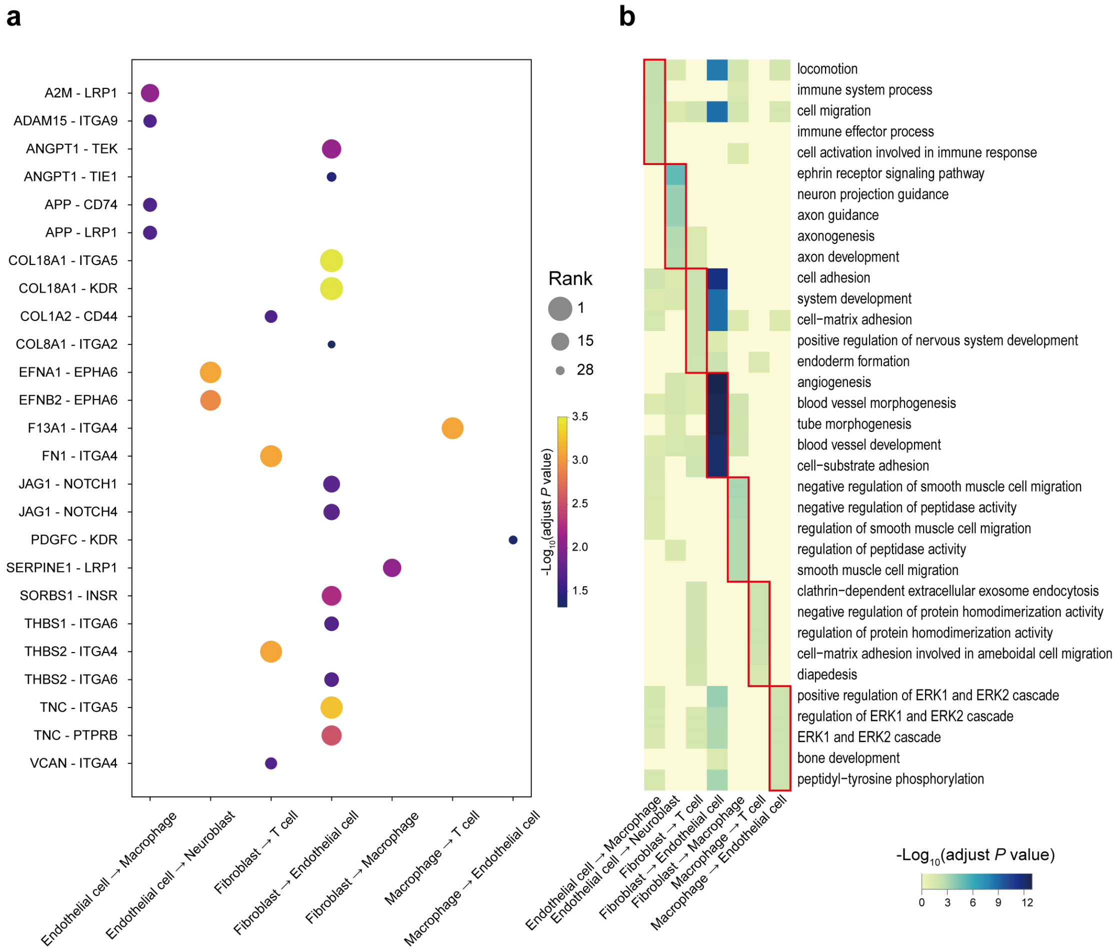

Response Fig 1. (Fig. 6). Cell-type-pair-specific functional modularity of *MYCN*-associated CCC signatures in the neuroblastoma dataset. a Dot plot summarizing *MYCN*-associated CCC signatures across different cell-type pairs in the neuroblastoma dataset. Each dot represents a ligand-receptor in CCC signatures, positioned by the corresponding sender–receiver cell-type pair. Dot size indicates the rank of the CCC signature based on association significance, while color denotes the strength of association calculated by $-\log_{10}$(adjusted P value). **b** Heatmap showing functional enrichment of *MYCN*-associated CCC signatures stratified by cell-type pairs. Rows represent enriched Gene Ontology (GO) biological process terms, and columns represent distinct sender-receiver cell-type pairs. Shades of blue in the heatmap are inversely proportional to the enrichment P values that were adjusted for multiple testing using the Benjamini-Hochberg method. Nonsignificant P values (> 0.05) are indicated in yellow. Red outlines highlight top five enriched functional modules for each cell-type-pair CCC signatures.

R1C2

2. Need to briefly discuss computational scalability for larger datasets or additional cancer types.

We thank the reviewer for this constructive suggestion. To address this concern, we have expanded the revised manuscript to evaluate both the applicability of Driver2Comm to additional cancer types and its computational scalability to larger datasets.

1. **Generalizability to additional cancer types.**

   We applied Driver2Comm to two additional cancer types (**a neuroblastoma dataset from Yu et al.** [24] **and an HPV-negative oral squamous cell carcinoma (OSCC) spatial transcriptomic dataset from Arora et al.** [25]).

   a) In the neuroblastoma dataset, Driver2Comm identified **29 *MYCN*-associated CCC signatures**, revealing that *MYCN*-driven communication patterns were organized into multiple **cell-type-specific functional modules** (see R1C1).
   b) In the OSCC dataset, Driver2Comm successfully identified driver gene-associated CCC signatures linked to *CDKN2A* and *EGFR*. Specifically, *CDKN2A*-associated CCC signatures were enriched in angiogenesis-related processes, including blood vessel and vasculature development (Response Fig. 2b, top panel). These findings suggested that loss of *CDKN2A* may reshape communication between tumor and stromal compartments to promote angiogenesis, consistent with prior studies showing that *CDKN2A* and its downstream products inhibit angiogenesis in SCC and other tumor types [26–28]. In contrast, *EGFR*-associated CCC signatures were enriched in keratinization and epithelial differentiation (Response Fig. 2b, bottom panel). These results imply that *EGFR* primarily influences the epithelial differentiation and keratinization programs, thereby maintaining aberrant keratinization and stem-like states, and thus promoting the tumor progression [29,30]. Additionally, these identified CCC signatures were also supported by spatially colocalization analysis (Response Figs. 2c and d).

   Taken together, these analyses demonstrate that Driver2Comm can be readily applied to diverse cancer types.

2. **Computational scalability to large datasets.**

   In the revised manuscript, we have added a brief discussion of computational scalability in the Discussion section. In this study, Driver2Comm was evaluated on datasets with up to 128 pseudo-samples. The primary computational bottleneck of Driver2Comm lies in the **frequent subnetwork mining** step. To address this, we employ the gSpan algorithm, an efficient and widely used approach for scalable frequent subgraph mining. The computational cost of gSpan is primarily determined by the choice of the minimum support threshold rather than the number of samples. Therefore, in practice, users can control computational cost by adjusting the minimum support threshold, providing a flexible trade-off between sensitivity and efficiency.

In summary, these results and discussions demonstrate that Driver2Comm is both generalizable across cancer types and computationally scalable to larger datasets. The corresponding discussion on computational scalability has been added to the revised **Discussion** section.

"*From a computational perspective, the primary cost of Driver2Comm lies in the frequent subnetwork mining step, which is implemented using the efficient gSpan algorithm. Importantly, computational cost is strongly influenced by the minimum support threshold, which in practice provides a flexible handle to balance sensitivity and efficiency as cohort size increases.*"

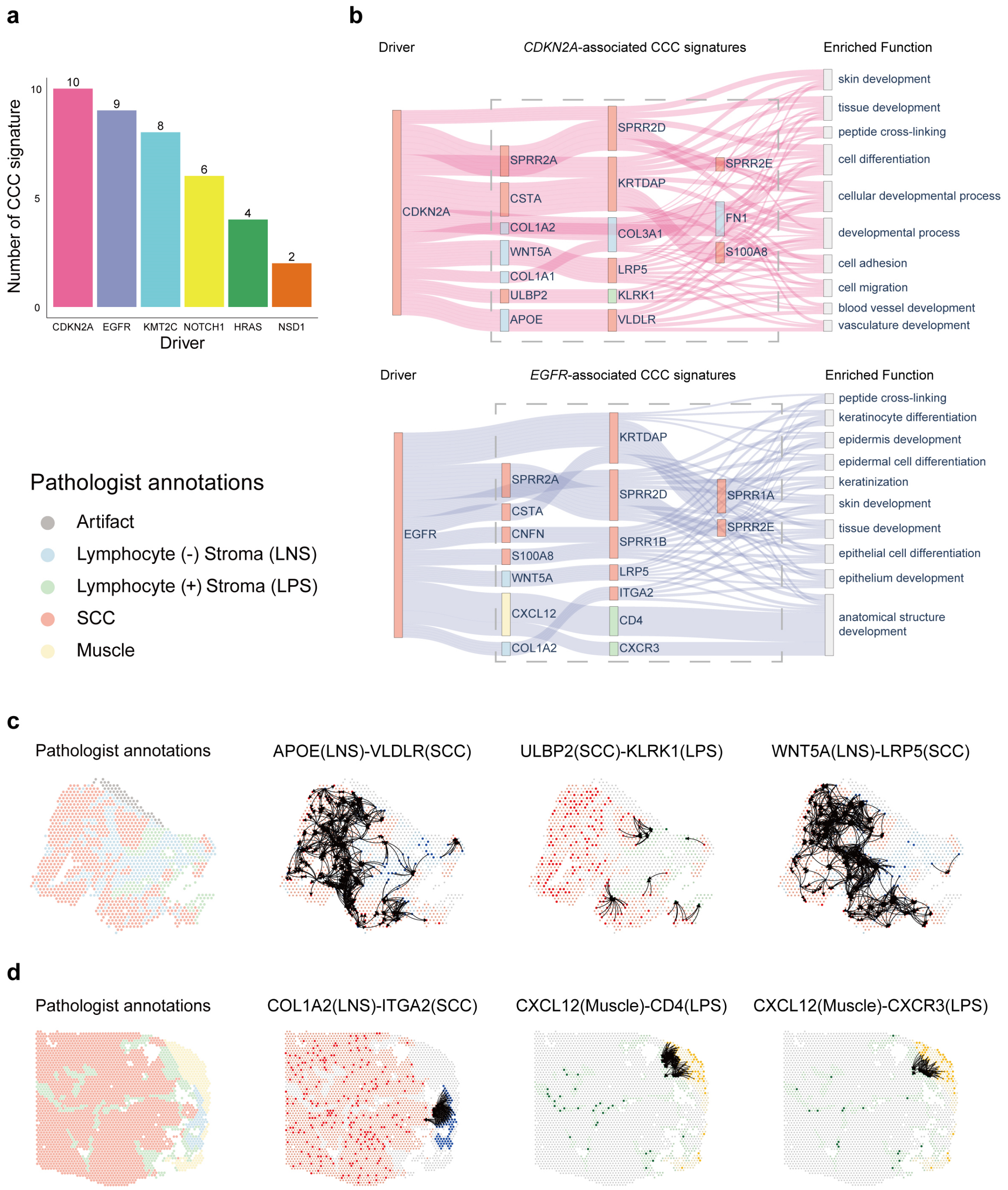

Response Fig. 2 (Fig. 7). Driver gene-associated CCC signatures in the OSCC dataset. a The

number of CCC signatures associated with drivers in the OSCC Visium dataset. **b** Sankey diagrams describing the correlation among driver genes, driver gene-associated CCC signatures and the corresponding top enriched functions. For nodes in driver and driver gene-associated CCC signatures, node labels are gene names. Node colors denote cell types: red for squamous cell carcinoma (SCC), blue for lymphocyte-negative stroma (LNS), green for lymphocyte-positive stroma (LPS), and yellow for muscle. For nodes in enriched function, node labels denote top 10 enriched function of genes in identified CCC signatures. Line width reflects the statistical significance of genes in CCC signatures enriched in the given functional term, as determined by Gene Ontology (GO) enrichment analysis (adjusted *P* value calculated using the Benjamini-Hochberg procedure). **c** Spatial maps of a representative *CDKN2A*-mutant OSCC sample. The first panel shows pathologist annotations. The subsequent panels show the activation of CCC signatures identified by Driver2Comm, including *APOE*(LNS) – *VLDLR*(SCC), *ULBP2*(SCC) – *KLRK1*(LPS), and *WNT5A*(LNS) – *LRP5*(SCC). **d** Spatial maps of a representative *EGFR*-mutant OSCC sample. The first panel shows pathologist annotations. The subsequent panels show the activation of CCC signatures identified by Driver2Comm, including *COL1A2*(LNS) – *ITGA2*(SCC), *CXCL12*(Muscle) – *CD4*(LPS), and *CXCL12*(Muscle) – *CXCR3*(LPS). In all spatial maps, edges highlight potential spots engaging in the corresponding CCC interactions.

Overall, Driver2Comm represents an important methodological advance with biological and clinical implications. The work provides a versatile resource for decoding cancer-immune crosstalk. I strongly recommend publication pending minor revisions.

We sincerely thank the reviewer for the positive and encouraging assessment of our work. We appreciate the recognition of Driver2Comm as a methodological advance with biological and clinical implications, as well as its potential as a versatile resource for decoding cancer–immune crosstalk. We have carefully addressed all minor revision suggestions raised by the reviewer and believe that these revisions have further strengthened the clarity, rigor, and impact of the manuscript. We are grateful for the reviewer’s time and constructive feedback.

Reviewer #2:

In this manuscript, the authors propose a software to analyze the associations between cancer driver genes and cell-cell communications. The idea is interesting and the examples are impressing. However, three major points should be addressed before acceptance for publication.

R2C1

1. First, it is an important but hard work to identify cancer driver mutations. The authors should explicitly assess how errors in identifying cancer driver genes affect the analytical results.

We thank the reviewer for raising this critical point. We fully agree that explicitly assessing how errors in identifying cancer driver genes affect the Driver2Comm results is necessary. In the revised manuscript, we systematically evaluated the robustness of Driver2Comm to inaccuracies in cancer driver gene annotation using two independent datasets with known mutated drivers (i.e., a breast cancer dataset from Wu et al. [31] and a neuroblastoma dataset from Yu et al. [24]).

Specifically, we simulated driver annotation errors by randomly introducing **2%–30% incorrect driver labels**, and compared the resulting driver gene-associated cell-cell communication (CCC) signatures with those obtained under the unperturbed (original) driver annotations. To quantitatively assess robustness under different levels of driver inaccuracy, we employed two complementary metrics.

(1) **Similarity index (SI)**[32] , which measures the consistency between CCC signatures identified under perturbed and unperturbed conditions:

$$SI = \frac{|A \cap B|}{\min\left(|A|, |B|\right)}$$

where A and B denote the sets of genes in driver gene-associated CCC signatures identified with and without driver perturbation, respectively.

(2) **Zero detection rate**, which quantifies the proportion of runs in which **no driver gene-associated CCC signatures were identified**. Importantly, this metric reflects **false-positive control**: when driver annotations are excessively corrupted, and the true association between driver genes and CCC signatures is disrupted, we expect Driver2Comm not to report spurious associations, but instead to return no result.

Our results show that:

- **Breast cancer dataset**: The identified CCC signatures remained highly consistent until the driver error rate exceeded 26% (Response Fig. 3a; **average SI > 0.8 at 26% driver error rate**). Meanwhile, when the error rate exceeded 16%, Driver2Comm progressively began to return no driver gene-associated CCC signatures. Notably, at a 30% error rate, the zero-detection rate reached 95%, indicating **strong suppression of false-positive discoveries** (Response Fig. 3b).
- **Neuroblastoma dataset**: CCC signatures remained robust across a wide range of perturbation levels (Response Fig. 3c; **average SI > 0.6 at a 30% driver error rate**). Similarly, once the error rate exceeded 16%, the zero-detection rate increased steadily with increasing perturbation (Response Fig. 3d), again demonstrating **effective false-positive control**.

Taken together, these results demonstrate that **Driver2Comm is highly robust to moderate levels of driver gene annotation errors**, maintaining consistent CCC

identification even under substantial perturbation. More importantly, Driver2Comm exhibits **excellent false-positive control**: when driver annotations become unreliable, the method tends to return no identified driver gene-associated CCC signatures instead of producing misleading results.

Full details of the robustness assessment are provided in **Supplementary Note 7**.

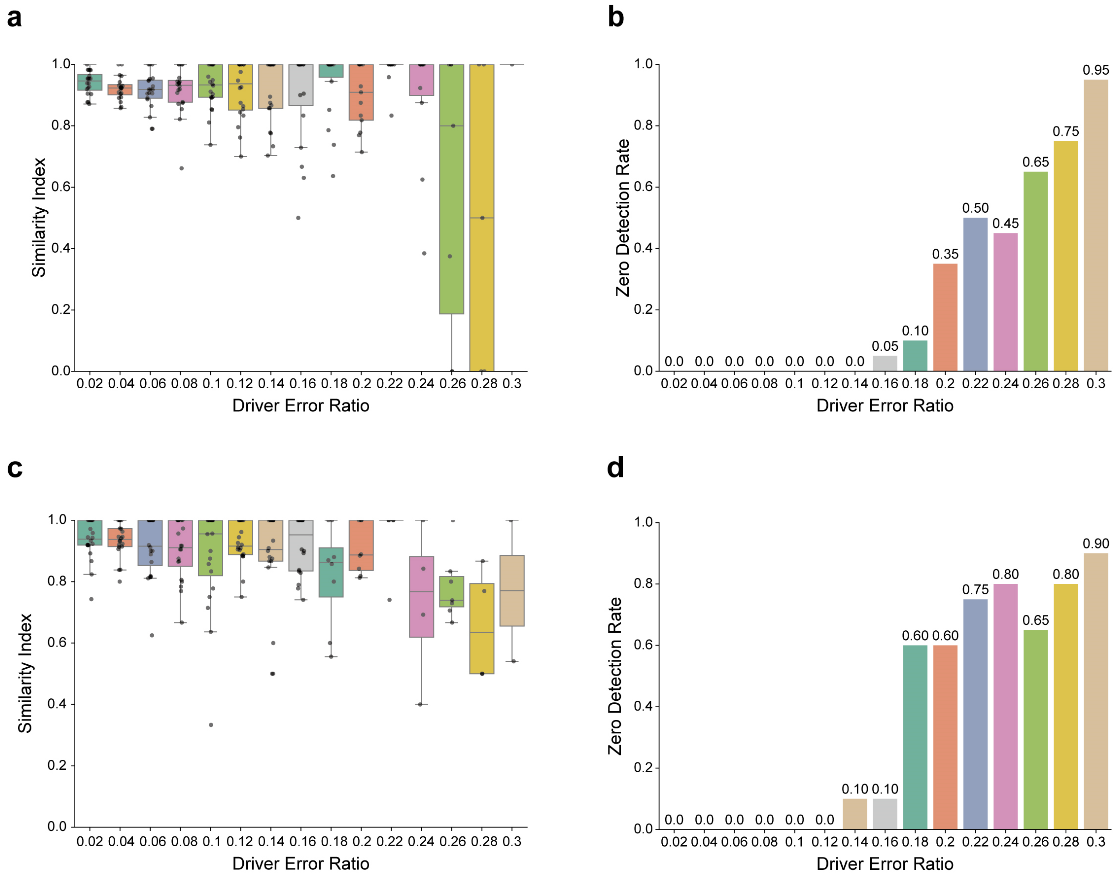

Response Fig. 3 (Supplementary Fig. 21). Robustness assessment of Driver2Comm to cancer driver gene annotation errors. (a-b) Robustness analysis on the breast cancer dataset. Random driver annotation errors (2%-30%) were introduced, and the identified driver gene-associated CCC signatures were compared with those obtained using the original driver labels. **(a)** Similarity Index between CCC signatures identified under perturbed and unperturbed conditions across different driver error ratios. Each box represents results from repeated random perturbations. Each dot represents of a run with driver gene-associated CCC signature detected. **(b)** Zero detection rate, defined as the proportion of runs in which no driver gene-associated CCC signatures were identified, under increasing driver error ratios. **(c-d)** Robustness analysis on the neuroblastoma dataset. **(c)** Similarity Index between CCC signatures identified under perturbed and unperturbed conditions across different driver error ratios. **(d)** Zero detection rate under increasing driver error ratios.

R2C2

2. Second, as spatial transcriptomics data are accomulating for tumor samples, the

authors should discuss and show how the software is applied to cancer spatial transcriptomics data. It is better to use cancer spatial transcriptomics data to validate the predictions.

We thank the reviewer for this insightful suggestion. In the revised manuscript, we have included a dedicated analysis demonstrating the applicability of Driver2Comm to cancer spatial transcriptomics data. Specifically, we applied Driver2Comm to an HPV-negative oral squamous cell carcinoma (OSCC) dataset profiled using the Visium V1 platform (comprising 12 patients with detailed pathological annotations). In this dataset, Driver2Comm successfully identified driver gene-associated CCC signatures for *CDKN2A*, *EGFR*, *KMT2C*, *NOTCH1*, *HRAS*, and *NSD1* (Response Fig. 4a). To validate the biological relevance of these predictions in a spatial context, we focused on the two most abundant *CDKN2A*- and *EGFR*-associated CCC signatures, and conducted two analyses:

1. **Functional characterization of CCC signatures:**
   CDKN2A-associated CCC signatures were enriched in angiogenesis-related processes, including blood vessel and vasculature development (Response Fig. 4b, top panel). These findings suggest that loss of *CDKN2A* may reshape communication between tumor and stromal compartments to promote angiogenesis, consistent with prior studies showing that *CDKN2A* and its downstream products inhibit angiogenesis in SCC and other tumor types [26–28]. In contrast, *EGFR*-associated CCC signatures were enriched in keratinization and epithelial differentiation (Response Fig. 4b, bottom panel). These results imply that *EGFR* primarily influences the epithelial differentiation and keratinization programs, thereby maintaining aberrant keratinization and stem-like states, and thus promoting the tumor progression [29,30].
2. **Spatial validation using Visium spot-level maps:**
   Spatial visualization of representative CCC signatures revealed clear co-localized interaction across tumor and stromal compartments, providing spatial support for the communication events predicted by Driver2Comm (Response Figs. 4c and d).

These new analyses are presented in the revised Results section. In summary, they demonstrate that Driver2Comm can be directly applied to spatial transcriptomics datasets and that its predicted driver gene-associated CCC signatures are supported by spatially co-localized patterns within tumor tissues.

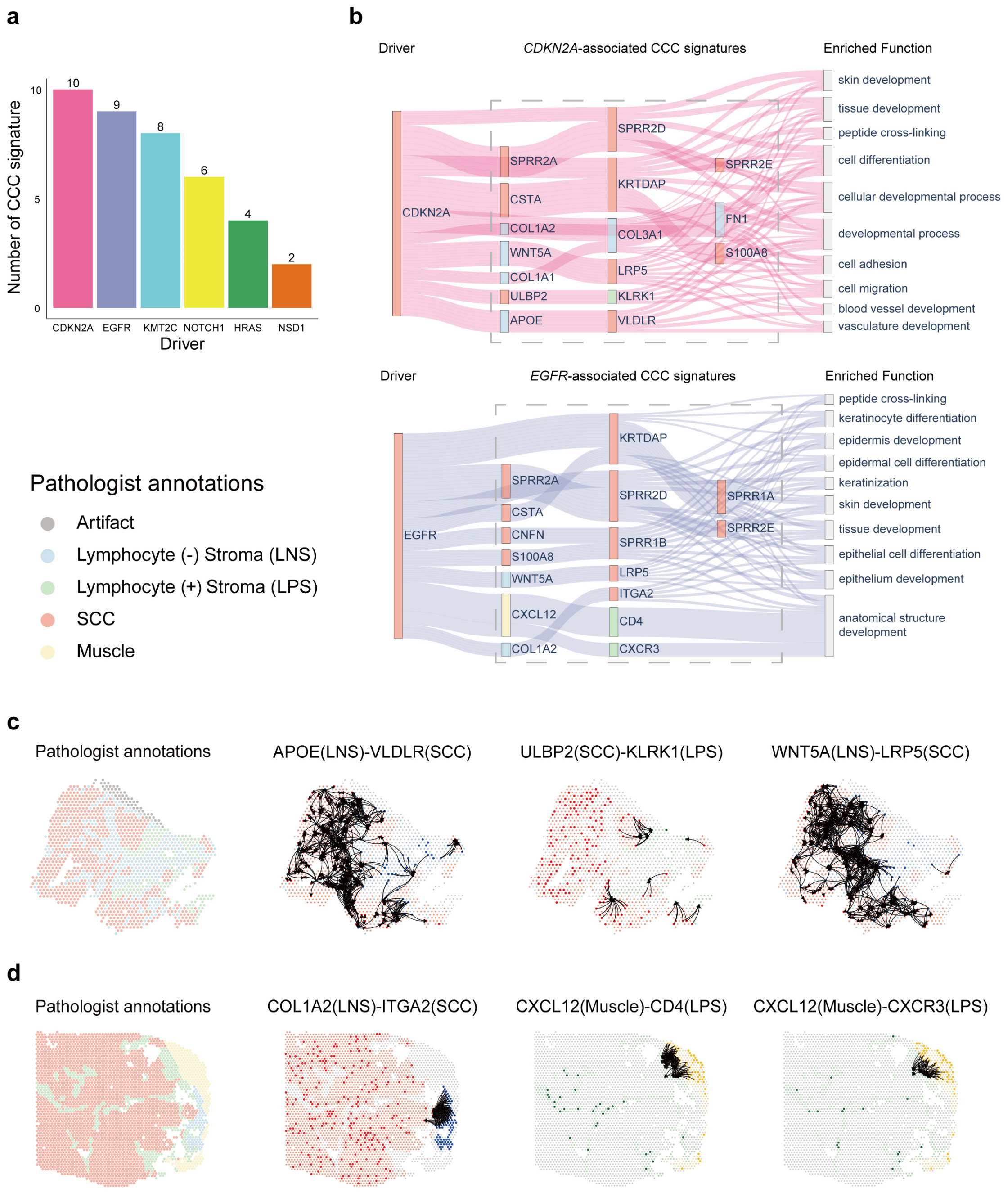

Response Fig. 4 (Fig. 7). Driver gene-associated CCC signatures in the OSCC dataset. a The number of CCC signatures associated with drivers in the OSCC Visium dataset. **b** Sankey diagrams describing the correlation among driver genes, driver gene-associated CCC signatures and the corresponding top enriched functions. For nodes in driver and driver gene-associated CCC signatures, node labels are gene names. Node colors denote cell types: red for squamous cell carcinoma (SCC), blue for lymphocyte-negative stroma (LNS), green for lymphocyte-positive stroma (LPS), and yellow for muscle. For nodes in enriched function, node labels denote top 10 enriched function of genes in identified CCC signatures. Line width reflects the statistical significance of genes in CCC signatures enriched in the given functional term, as determined by Gene Ontology (GO) enrichment analysis (adjusted *P* value calculated using the Benjamini-Hochberg procedure). **c** Spatial maps of a representative *CDKN2A*-mutant OSCC sample. The first panel shows pathologist annotations. The subsequent panels show the activation of CCC signatures

identified by Driver2Comm, including *APOE*(LNS)-*VLDLR*(SCC), *ULBP2*(SCC)-*KLRK1*(LPS), and *WNT5A*(LNS)-*LRP5*(SCC). **d** Spatial maps of a representative *EGFR*-mutant OSCC sample. The first panel shows pathologist annotations. The subsequent panels show the activation of CCC signatures identified by Driver2Comm, including *COL1A2*(LNS)-*ITGA2*(SCC), *CXCL12*(Muscle)-*CD4*(LPS), and *CXCL12*(Muscle)-*CXCR3*(LPS). In all spatial maps, edges highlight potential spots engaging in the corresponding CCC interactions.

R2C3

3. Third, it is better to discuss whether cell-cell communication is helpful for identifying cancer driver genes.

We appreciate the reviewer for the insightful suggestion. We agree that discussing whether CCC is helpful for identifying cancer driver genes would add valuable conceptual depth to our work. We have accordingly added a new discussion to elaborate on this important point.

Specifically, we now discuss the bidirectional relationship between CCC and cancer driver genes. While numerous studies demonstrate that cancer driver gene reshaped the CCC pattern in the tumor microenvironment (TME)[1,33–36], accumulating experimental evidence suggests that extrinsic CCC can also shape the selective landscape of cancer evolution, thereby influencing which genetic alterations are retained and clonally expanded. For example, immunoediting processes and specific CCC in the TME can exert selective pressure that favors clones with particular mutations[37]. Notably, astrocyte-derived exosomal miR-19a has been shown to downregulate *PTEN* in cancer cells, facilitating brain metastasis[38], and acquired genetic mutations in the IFN-γ signaling pathway have been identified in melanoma tumors resistant to immunotherapy[39] .

Taken together, these findings suggest that the CCC landscape contributes to the selective advantage of specific genomic alterations. We therefore believe that integrating CCC features into driver gene discovery frameworks holds great potential for prioritizing functionally important mutations.

Full discussion of this points has been added to the Introduction section:

"Conversely, extrinsic CCC signals can also shape the selective landscape of cancer evolution, influencing which genetic alterations are retained and clonally expanded. Specifically, immune cell-mediated interactions, such as IFN-γ signaling, PD-L1 engagement, or antigen presentation, can eliminate immunogenic subclones while favoring those harboring mutations that enable immune escape[13-15]. These observations imply that extrinsic communication signals not only respond to, but also shape, the selection of driver gene alterations."

Reviewer #3:

Understanding how the cancer cell intrinsic driver genes interact with -extrinsic cell-cell communication (CCC) events in the TME is important for studying the cancer progression and identifying microenvironment-related targets. The authors present a computational framework Driver2Comm to identify intrinsic-extrinsic (IE) pathways that functionally connect cancer cell driver genes with their associated CCC signatures. This framework integrates the authors' previously developed CytoTalk, frequent subnetwork mining and association testing to identify cancer driver-associated CCC signatures. The authors demonstrated the performance of Driver2Comm using three distinct cancer datasets. Below are specific comments.

R3C1

1. There are several CCC inference methods that consider both intercellular CCC and intracellular downstream response, such as scMLnet and CellCall. Are these methods applicable to identify cancer driver-associated CCC signatures with the help of association testing, which can be also used to perform the survival analysis. It will be great to clarify the differences in biological discovery between Driver2Comm and other CCC methods.

We thank the reviewer for this valuable suggestion. Indeed, CCC inference methods that incorporate both intercellular CCC and intracellular downstream response, such as scMLnet and CellCall, is applicable to identify cancer driver-associated CCC signatures by the general Driver2Comm framework. The resulting CCC signatures can also be used to perform the survival analysis.

However, conceptually, methods like scMLnet and CellCall unable to identify intrinsic-extrinsic (IE) pathways, because they do not model **upstream signaling pathways** of intercellular CCC. In contrast, CytoTalk reconstructs full signaling networks including both upstream pathways of ligands and downstream pathways of receptors, enabling Driver2Comm to establish explicit links between driver genes and CCC signatures.

To clarify the differences in biological discovery of different CCC inference methods, we systematically compared CytoTalk-based and scMLnet-based Driver2Comm on two datasets (PDAC and breast cancer datasets). Differentially expressed genes (DEGs) stratified by driver status were also included as a baseline in survival analysis. CellCall was excluded due to its computational infeasibility in multi-sample settings. The results showed that:

1. **In the PDAC dataset**: scMLnet-based Driver2Comm did not identify any *BRCA1*-associated CCC signatures, thus comparisons focused on *AKT2*-associated CCC signatures. Notably, the CCC signatures identified by the two methods showed no overlap (Response Fig. 5a). CytoTalk-based Driver2Comm

predominantly detected intercellular interactions, whereas scMLnet-based Driver2Comm mainly identified intracellular regulatory interactions within macrophages (Response Fig. 5b). Functional enrichment analysis showed that CytoTalk-specific CCC signatures uniquely involved transmembrane signaling and immune-related pathways, while scMLnet-specific terms were enriched for RNA/DNA metabolic processes (Response Fig.5c). Importantly, CytoTalk-based Driver2Comm identified a substantially higher proportion of CCC signatures with significant prognostic effects (Response Fig. 5d), and CytoTalk-derived signatures were more strongly associated with patient survival than both DEGs and scMLnet-derived signatures (Response Fig. 5e).

2. **In the breast cancer dataset**: Consistent with the PDAC results, the two methods yielded markedly different *ESR1*-associated CCC signatures (Response Fig. 6a). CytoTalk-based Driver2Comm identified intercellular interactions primarily between macrophages and tumor cells, whereas scMLnet-based Driver2Comm mainly captured intracellular regulatory signals within CD8+ T cells (Response Fig. 6b). CytoTalk-specific functional terms were enriched for chemokine regulation and transmembrane receptor signaling, while scMLnet-specific terms involved miRNA regulation, RNA metabolism, and apoptotic pathways (Response Fig. 6c). Survival analysis show that CytoTalk-based Driver2Comm identified substantially more proportion of CCC signatures that with significant prognostic effect. Again, CytoTalk-based Driver2Comm detected more CCC signatures with significant prognostic value (Response Fig. 6d), and these signatures showed stronger associations with survival than both DEGs and scMLnet-derived signatures (Response Fig. 6e).

Overall, our comparative analyses show that:

- **CytoTalk-based Driver2Comm** preferentially identifies **intercellular pathways**, which more directly reflect cell-cell interactions in the tumor microenvironment.
- **scMLnet-based Driver2Comm** tends to capture **intracellular regulatory programs**, which are less directly linked to intercellular signaling.
- **CytoTalk-based Driver2Comm** consistently identifies **more CCC signatures with significant prognostic effects** than other methods across datasets.

Therefore, we found that CytoTalk provides particular advantages within the Driver2Comm framework for reconstructing IE pathways and for identifying CCC signatures with consistent prognostic relevance across datasets.

Full details of the comparison of CCC inference methods in Driver2Comm is provided in **Supplementary Note 6**.

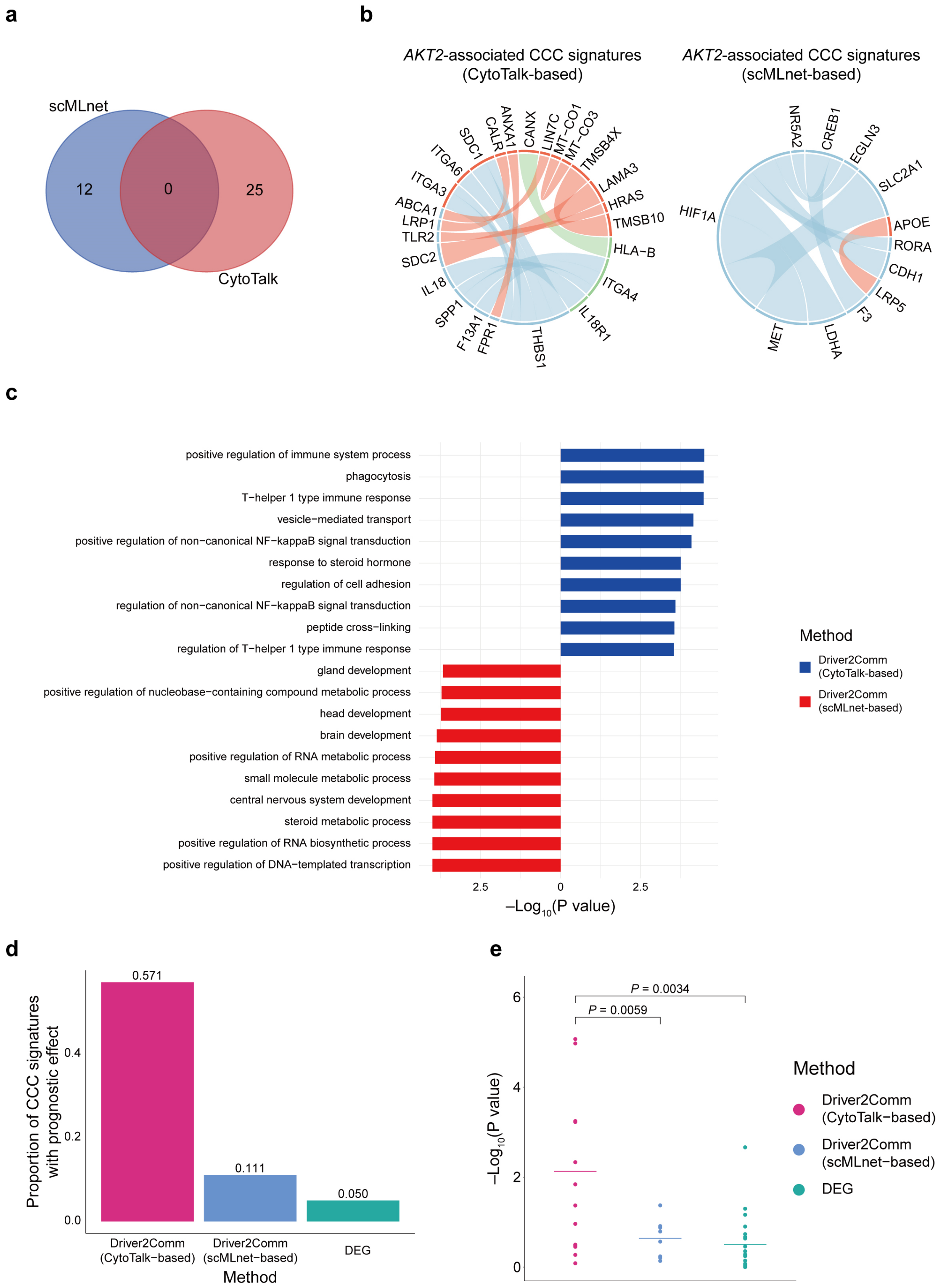

Response Fig. 5 (Supplementary Fig. 19) Performance comparison of CCC inference methods used in Driver2Comm on the PDAC dataset. a Venn diagram showing the gene-level overlap of *AKT2*-associated CCC signatures identified by scMLnet-based and CytoTalk-based Driver2Comm. **b** Chord diagram visualization of *AKT2*-associated CCC signatures identified by CytoTalk-based Driver2Comm (left) and scMLnet-based Driver2Comm (right). Node represent genes, and colors indicate cell types: red for tumor cells, blue for macrophages, and green for CD8+ T cells. Edge colors indicate the sender cell type. Edge width reflected the association

strength between cancer driver and corresponding driver gene-associated CCC signatures. **c** Top uniquely enriched biological processes for CCC signatures identified by CytoTalk-based (blue) and scMLnet-based Driver2Comm (red). **d** Proportion of identified CCC signatures with significant prognostic effects across methods. **e** Performance evaluated by $-\log_{10}(P \text{ value})$ of log-rank test for survival associations of CCC signatures across methods or DEGs. Each point corresponds to the performance on an identified CCC signatures or DEGs, with horizontal bars indicating the mean performance across all methods. *P* values for comparison were calculated using one-sided *t*-tests.

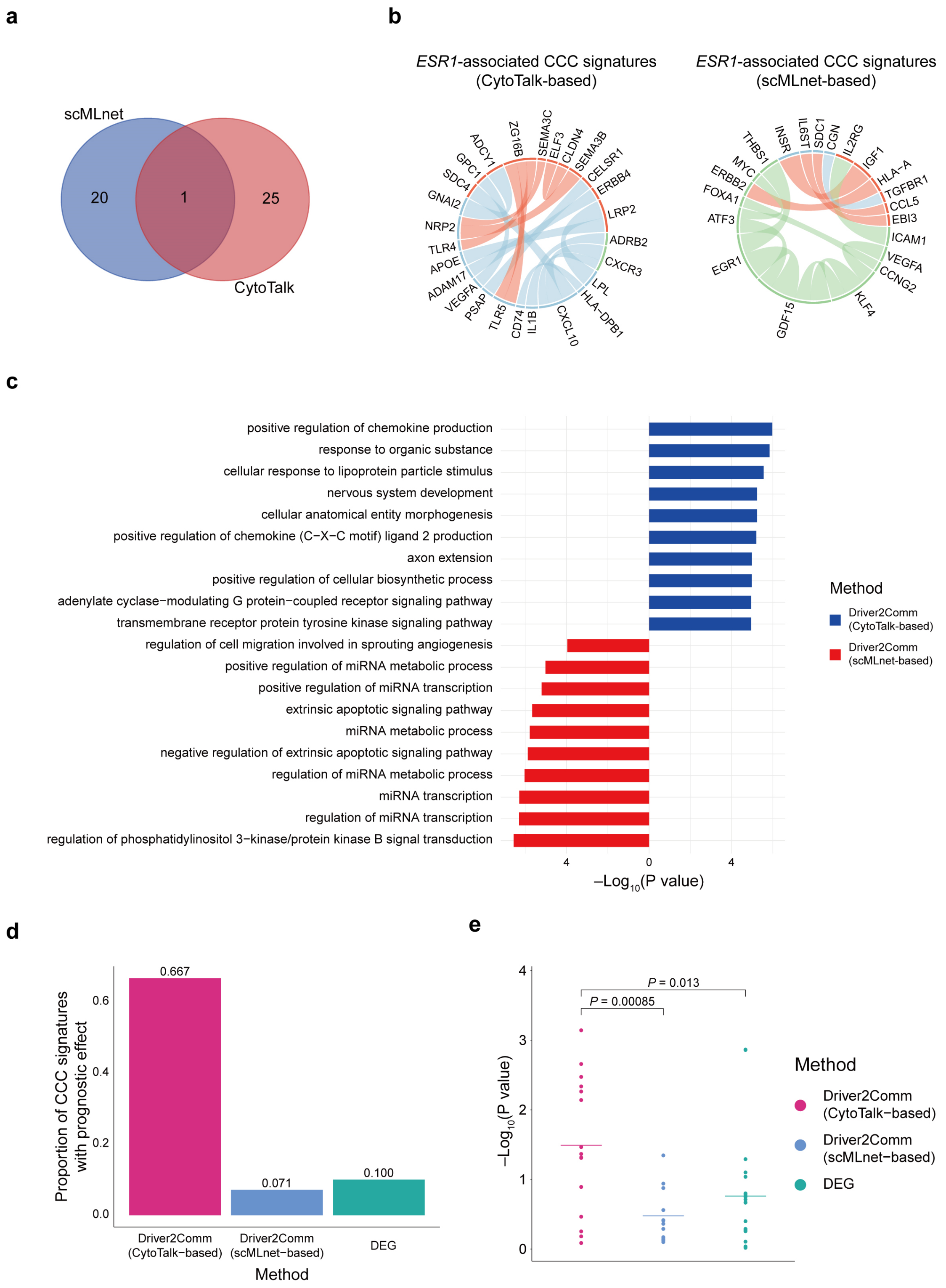

Response Fig. 6 (Supplementary Fig. 20) Performance comparison of CCC inference methods used in Driver2Comm on the breast cancer dataset from Wu et al.. a Venn diagram showing the gene-level overlap of *ESR1*-associated CCC signatures identified by scMLnet-based and CytoTalk-based Driver2Comm. **b** Chord diagram visualization of *ESR1*-associated CCC signatures identified by CytoTalk-based Driver2Comm (left) and scMLnet-based Driver2Comm (right). Node represent genes, and colors indicate cell types: red for tumor cells, blue for macrophages, and green for CD8+ T cells. Edge colors indicate the sender cell type. Edge width

reflected the association strength between cancer driver and corresponding driver gene-associated CCC signatures. **c** Top uniquely enriched biological processes for CCC signatures identified by CytoTalk-based (blue) and scMLnet-based Driver2Comm (red). **d** Proportion of identified CCC signatures with significant prognostic effects across methods. **e** Performance evaluated by $-\log_{10}$ (*P* value) of log-rank test for survival associations of CCC signatures across methods or DEGs. Each point corresponds to the performance on an identified CCC signatures or DEGs, with horizontal bars indicating the mean performance across all methods. *P* values for comparison were calculated using one-sided *t*-tests.

R3C2

2. How did the frequent subnetwork mining method ensure that the identified subnetwork consists of both intrinsic driver genes and extrinsic CCC?

We thank the reviewer for raising this question. We would like to clarify that the frequent subnetwork mining procedure was **only used to extract recurrent and robust CCC subnetworks**, and is not designed to ensure that intrinsic driver genes and extrinsic CCC appear in the same subnetwork. Instead, the integration of intrinsic driver genes and extrinsic CCC occurs **only on the IE pathway identification step**, where the driver gene is connected to its associated CCC signature through the CytoTalk-built candidate signaling network. This signaling network contains both driver gene and associated CCC signatures, which enables the construction of IE pathways.

Specifically, Driver2Comm achieved this through the following workflow. First, frequent subnetwork mining was performed on the CytoTalk-built multi-cell-type communication (MCTC) networks to extract recurrent and robust CCC subnetworks, which serve as candidate extrinsic CCC signatures. Second, association testing was conducted to identify CCC signatures that were statistically associated with each cancer driver gene across samples.

After driver gene-associated CCC signatures were identified, IE pathway reconstruction was performed. Because cancer driver genes were not guaranteed to be included in the CCC-focused MCTC network, we expanded the MCTC network using the CytoTalk-built intracellular network as a reference. Starting from nodes in the MCTC network, first-order neighbors in the intracellular network were iteratively incorporated until the driver gene was reached. IE pathways were then identified as the shortest paths connecting the driver gene and its associated CCC signatures within the expanded MCTC network.

Thus, **frequent subnetwork mining identifies robust CCC signatures**, while **IE pathway reconstruction guarantees explicit linking between driver genes and their associated CCC signatures**. We have revised the workflow of IE pathway identification for better clarification in the corresponding Methods section.

"*We identified IE pathways using the following procedure. In the previous step, driver-associated CCC signatures were extracted from the MCTC network. Because the MCTC network was centered around ligand–receptor pairs [34], cancer driver genes were not guaranteed to be included in this network. To explicitly connect cancer cell-intrinsic driver genes with -extrinsic CCC signatures, we expanded the MCTC network to incorporate the driver gene using the CytoTalk-built intracellular network. Specifically, for each node in the MCTC network, we iteratively incorporated its first-order neighbors from the intracellular network until the driver gene was reached. Finally, the shortest paths between the driver gene and its associated CCC signatures in the expanded MCTC networks were identified as the IE pathways.*"

R3C3 (a)

3. (a) It is important to compare the performance with the baseline methods such as simply using the cancer drivers or DEGs.

We thank the reviewer for this constructive suggestion. In the revised manuscript, we added a comprehensive comparison of survival relevance among CytoTalk-based Driver2Comm (method applied in this study), scMLnet-based Driver2Comm, and top 20 driver-associated DEGs as the baseline method, with the number of genes chosen to be comparable to the typical number of CCC signatures identified by Driver2Comm, thereby reducing potential bias arising from differences in candidate set size. We evaluated the survival performance of different methods from two complementary perspectives: (a) the **proportion of identified CCC signatures or genes with significant prognostic effects**, which reflects the recall of prognostically relevant signals; and (b) the **distribution of $-\log_{10}$(*P* values)** from log-rank tests, which assesses the overall strength of survival associations captured by each method.

The results showed that:

1. In the PDAC dataset, **CytoTalk-based Driver2Comm consistently achieved the best performance for both *AKT2*- and *BRCA1*-driven cohorts**. Specifically, in the *AKT2*-driven PDAC cohort, CytoTalk-based Driver2Comm identified a significantly higher proportion of prognostically relevant CCC signatures and exhibited significantly stronger overall survival associations than scMLnet-based Driver2Comm and DEGs (Response Figs.7a and b). In the *BRCA1*-driven PDAC cohort, CytoTalk-based Driver2Comm showed superior average survival associations compared with DEGs, albeit without statistical significance (Response Figs.7c and d) scMLnet-based Driver2Comm was not included in this comparison because it did not identify any *BRCA1*-associated CCC signatures.
2. In the breast cancer dataset, CytoTalk-based Driver2Comm also achieved the best performance in the *ESR1*-driven cohort. Both the proportion of prognostically significant CCC signatures and the overall survival associations were significantly

higher than those obtained using scMLnet-based Driver2Comm or DEGs (Response Figs.7e and f).

Taken together, these results demonstrate that Driver2Comm identifies CCC signatures with stronger and more consistent survival relevance than baseline strategies based on driver-associated DEGs alone.

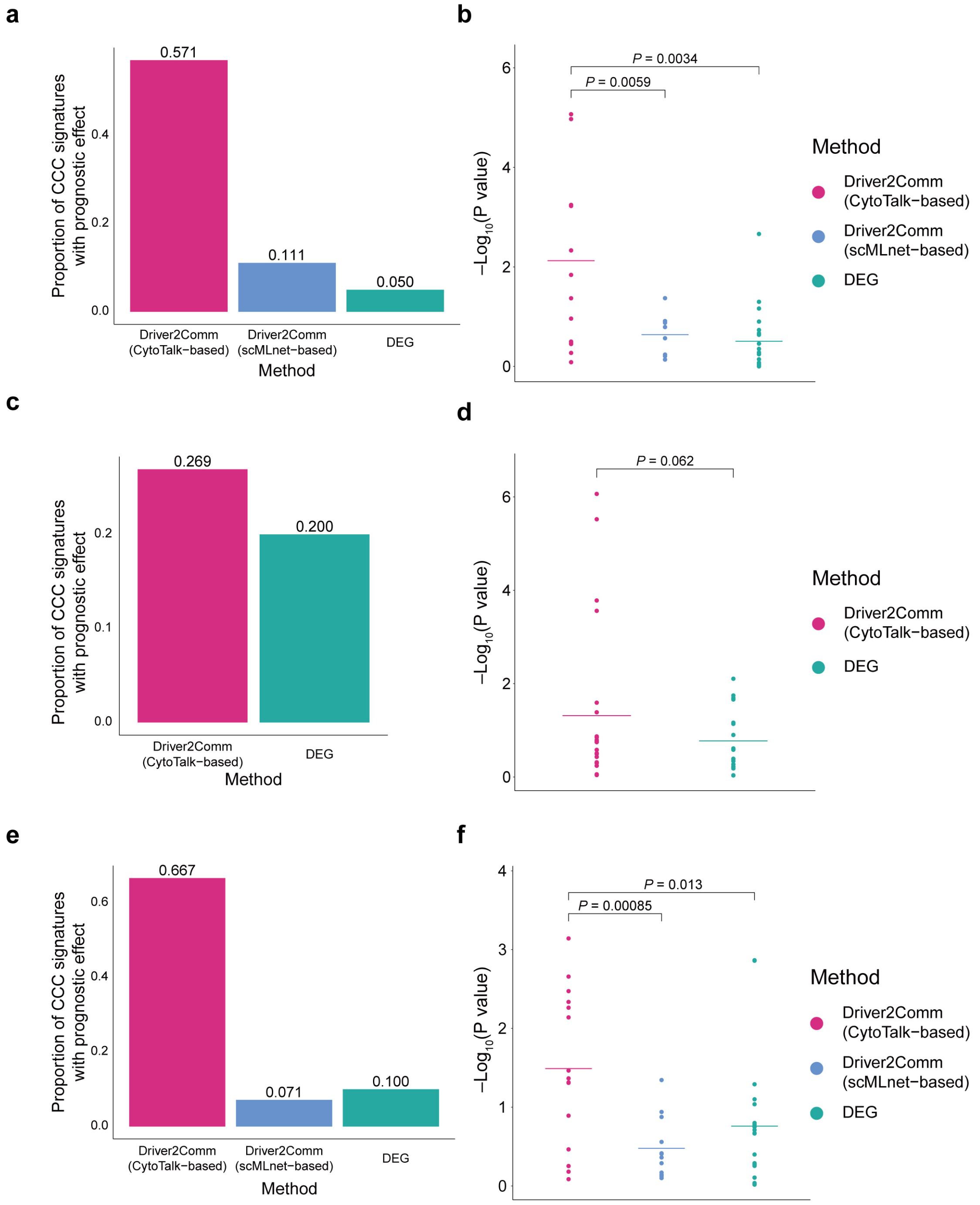

Response Fig. 7. Survival relevance comparison between Driver2Comm and baseline methods. a, Proportion of identified *AKT2*-associated CCC signatures with significant prognostic effects across methods. **b** Distribution of $-\log_{10}$ (*P* values) from log-rank tests evaluating survival

associations of *AKT2*-associated CCC signatures across methods or *AKT2*-associated differentially expressed genes (DEGs). **c** Proportion of identified *BRCA1*-associated CCC signatures with significant prognostic effects across methods. **d** Distribution of $-\log_{10}$ (*P* value) from log-rank tests for survival associations of *BRCA1*-associated CCC signatures across methods or *BRCA1*-associated DEGs. **e** Proportion of identified *ESR1*-associated CCC signatures with significant prognostic effects across methods. **f** Distribution of $-\log_{10}$ (*P* value) from log-rank tests for survival associations of *ESR1*-associated CCC signatures across methods or *ESR1*-associated DEGs. In panels **b**, **d**, and **f**, each point represents the survival association of an individual CCC signature or DEG, and horizontal bars indicate the mean performance for each method. Statistical comparisons between methods were performed using one-sided *t* tests.

R3C3 (b)

(b) In addition, the difference among the different lines in Figure 3 does not seem significant. The authors should add more descriptions of the figure legends. For example, what is the meaning of different colors in Figure 3f? How did the low-rank test perform? Please clarify the comparison groups for the indicated p-value?

We thank the reviewer for this helpful suggestion. We have revised the figure legends of Figure 3 to more clearly describe the meaning of colors, the comparison groups used in the survival analyses, and the interpretation of statistical tests. Additional clarifications are provided below.

First, regarding the comment that differences among survival curves do not seem significant, we used a **multi-group log-rank test**, which evaluates whether all stratified patient groups share the same survival distribution. The significant result (*P* values <0.05) indicates that at least one subgroup exhibits a survival pattern distinct from the others, demonstrating the overall prognostic relevance of the *ESR1*-associated CCC signature. Pairwise log-rank tests were not performed, as our goal was to assess the global prognostic relevance of each CCC signature rather than compare every pair of subgroups. The relatively similar shapes of some Kaplan-Meier curves reflect moderate effect sizes, while statistical significance is supported by the large METABRIC ER+ cohort size (n = 1,196).

Second, we have updated the figure legend to explicitly define the color scheme used in Figure 3f-i. Specifically, Figure 3f represents a **combinatorial survival stratification** based on an *ESR1*-associated CCC signature involving *CXCR3* in CD8+ T cells, *CXCL10* in macrophages, and *SDC4* in tumor cells. For each component, patients were stratified into high and low groups based on the median expression (or subtype abundance), resulting in all possible combinations of high/low states across the three components. Each colored curve in Figure. 3f corresponds to one such combinatorial pattern, as now explicitly indicated in the legend.

Third, we clarify that the reported *P* values were obtained using the multi-group log-rank test and the comparison group involved all stratified patient groups simultaneously. This is now explicitly stated in both the figure legend.

Taken together, these revisions improve the clarity and interpretability of all figure related to survival analysis and ensure that all statistical comparisons are self-contained and transparent to readers.

"*Patients were stratified according to the expression of genes involved in ESR1-associated CCC signatures. For each gene in a given CCC signature, patients were categorized into highly-expressed and lowly-expressed groups based on the median expression value. All possible combinations of high/low states across the involved genes in the CCC signatures were considered, and each colored curve represents one such combinatorial expression pattern, as indicated in the legend. Survival differences among all stratified groups were evaluated using a multi-group log-rank test, and the reported P values correspond to the global comparison across all groups within each panel.*"

R3C4

4. It is not very common to study CCC among only two cell types? It is better if the authors can show one example with common number of cell types in the dataset.

We thank the reviewer for this insightful suggestion. We fully agree that CCC in the TME typically involves multiple interacting cell types, and that demonstrating the availability of Driver2Comm on datasets with common number of cell types is essential. In the original manuscript, we focused on cancer cells, macrophages, and CD8+ T cells, **as the central roles of macrophages and CD8+ T cells in innate and adaptive immunity**, respectively. In the revised manuscript, we have therefore **added a comprehensive analysis using a neuroblastoma dataset from Yu et al.** [24]**, comprising eight major cell types**. In this analysis, we constructed a MCTC network including all eight cell types and applied Driver2Comm without restricting the analysis to any predefined cell-type pairs.

Driver2Comm identified 29 *MYCN*-associated CCC signatures, which exhibited multiple **cell-type-pair-specific functional modules**. Specifically,

- **Fibroblast-endothelial cell** communication emerged as the dominant *MYCN*-associated CCC signatures (Response Fig. 8a), which was enriched for angiogenesis-related processes (Response Fig. 8b), aligned with the previous findings linking *MYCN* amplification to increased vascularity in neuroblastoma [14–16].
- **Endothelial cell-macrophage** interactions revealed distinct immune regulatory programs, including immune cell migration, locomotion, and immune system

processes (Response Fig. 8b), consistent with the known role of endothelial cells in myeloid cell recruitment and positioning within the TME [17,18].

- Only limited **T cell-involving** *MYCN*-associated CCC signatures were observed (Response Fig. 8a), which may reflect immunologically "cold" status in the TME in *MYCN* amplification neuroblastoma[19–21].
- **Fibroblast-to-endothelial cell** interactions were enriched for the ephrin receptor signaling pathway (Response Fig. 8b), which was well known for its roles in neural and vascular development. Prior studies have linked ephrin signaling components with *MYCN* amplification and prognosis in neuroblastoma [22,23].

These results demonstrate that Driver2Comm is capable of capture **complex, higher-order CCC architectures** involving multiple interacting cell types, showing that Driver2Comm is fully general and readily applicable to datasets with **a common or large number of cell types**.

Full details of neuroblastoma analysis are provided in the Results section under the heading: **"*MYCN*-associated CCC signatures exhibit cell-type-pair-specific functional modularity"**.

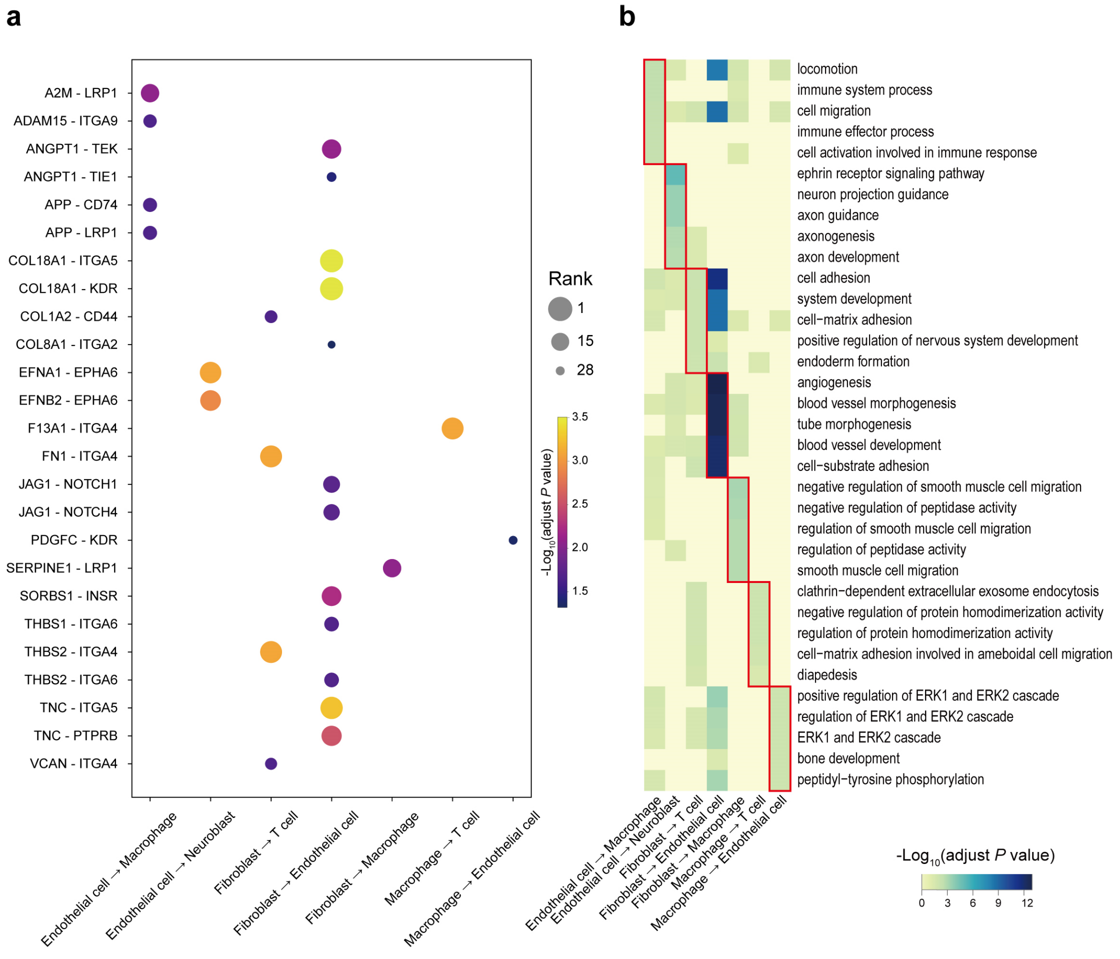

Response Fig. 8 (Fig. 6). Cell-type-pair-specific functional modularity of *MYCN*-associated CCC signatures in the neuroblastoma dataset. a Dot plot summarizing *MYCN*-associated CCC

signatures across different cell-type pairs in the neuroblastoma dataset. Each dot represents a ligand-receptor in CCC signatures, positioned by the corresponding sender–receiver cell-type pair. Dot size indicates the rank of the CCC signature based on association significance, while color denotes the strength of association calculated by $-\log_{10}$(adjusted *P* value). **b** Heatmap showing functional enrichment of *MYCN*-associated CCC signatures stratified by cell-type pairs. Rows represent enriched Gene Ontology (GO) biological process terms, and columns represent distinct sender-receiver cell-type pairs. Shades of blue in the heatmap are inversely proportional to the enrichment *P* values that were adjusted for multiple testing using the Benjamini-Hochberg method. Nonsignificant *P* values (> 0.05) are indicated in yellow. Red outlines highlight top five enriched functional modules for each cell-type-pair CCC signatures.

R3C5

5. Please clarify the meaning of the line width between CCC signatures and the enriched functions in the Sankey diagram.

We appreciate the reviewer's careful observation. The width of the connecting lines between CCC signatures and enriched functions in the Sankey diagram reflected the statistical significance of functional enrichment. Specifically, a wider line indicates that the corresponding CCC signature is more significantly enriched in the given functional category, as determined by Gene Ontology (GO) enrichment analysis (adjusted *P* value calculated using the Benjamini–Hochberg procedure). In other words, the line width is inversely proportional to the adjusted *P* value; the smaller the *P* value, the thicker the line. This visual representation is intended to highlight which functional processes are more strongly associated with each CCC signature. We have added this explanation to the figure legend in the revised manuscript to improve clarity.

"*The line width reflected the statistical significance of CCC signature enriched in the given functional category, as determined by Gene Ontology (GO) enrichment analysis (adjusted P value calculated using the Benjamini-Hochberg procedure). It is inversely proportional to the adjusted P value.*"

References

1. Liao, W. *et al.* KRAS-IRF2 Axis Drives Immune Suppression and Immune Therapy Resistance in Colorectal Cancer. *Cancer Cell* **35**, 559-572.e7 (2019).
2. Madeddu, C. *et al.* EGFR-Mutated Non-Small Cell Lung Cancer and Resistance to Immunotherapy: Role of the Tumor Microenvironment. *IJMS* **23**, 6489 (2022).
3. Misetic, H., Keddar, M. R., Jeannon, J.-P. & Ciccarelli, F. D. 23 Genome Med Mechanistic insights into the interactions between cancer drivers and the tumour immune microenvironment. *Genome Med* **15**, 40 (2023).
4. Ji, S., Shi, Y. & Yin, B. Macrophage barrier in the tumor microenvironment and potential clinical applications. *Cell Commun Signal* **22**, 74 (2024).
5. Du, Y. *et al.* Potential crosstalk between SPP1 + TAMs and CD8 + exhausted T cells promotes an immunosuppressive environment in gastric metastatic cancer. *J Transl Med* **22**, 158 (2024).
6. Li, M., Jiang, P., Wei, S., Wang, J. & Li, C. The role of macrophages-mediated communications among cell compositions of tumor microenvironment in cancer progression. *Front. Immunol.* **14**, 1113312 (2023).
7. Raskov, H., Orhan, A., Christensen, J. P. & Gögenur, I. Cytotoxic CD8+ T cells in cancer and cancer immunotherapy. *Br J Cancer* **124**, 359–367 (2021).
8. Wang, L. *et al.* Pan-cancer analysis reveals tumor-associated macrophage communication in the tumor microenvironment. *Exp Hematol Oncol* **10**, 31 (2021).
9. Berg, R. E., Cordes, C. J. & Forman, J. Contribution of CD8+ T cells to innate immunity: IFN-γ secretion induced by IL-12 and IL-18. *Eur. J. Immunol.* **32**, 2807–

2816 (2002).

10. Maimela, N. R., Liu, S. & Zhang, Y. Fates of CD8+ T cells in Tumor Microenvironment. *Computational and Structural Biotechnology Journal* **17**, 1–13 (2019).

11. Mantovani, A., Allavena, P., Marchesi, F. & Garlanda, C. Macrophages as tools and targets in cancer therapy. *Nat Rev Drug Discov* **21**, 799–820 (2022).

12. Duan, Z. & Luo, Y. Targeting macrophages in cancer immunotherapy. *Sig Transduct Target Ther* **6**, 127 (2021).

13. Binanewies, M. *et al.* Understanding the tumor immune microenvironment (TIME) for effective therapy. *Nat Med* **24**, 541–550 (2018).

14. Meitar, D., Crawford, S. E., Rademaker, A. W. & Cohn, S. L. Tumor angiogenesis correlates with metastatic disease, N-myc amplification, and poor outcome in human neuroblastoma. *J Clin Oncol* **14**, 405–414 (1996).

15. Eggert, A. *et al.* High-level expression of angiogenic factors is associated with advanced tumor stage in human neuroblastomas. *Clin Cancer Res* **6**, 1900–1908 (2000).

16. Chesler, L. *et al.* Malignant Progression and Blockade of Angiogenesis in a Murine Transgenic Model of Neuroblastoma. *Cancer Res* **67**, 9435–9442 (2007).

17. McCoy, M. G. *et al.* Endothelial TLR2 promotes proangiogenic immune cell recruitment and tumor angiogenesis. *Science Signaling* **14**, eabc5371 (2021).

18. Amersfoort, J., Eelen, G. & Carmeliet, P. Immunomodulation by endothelial cells — partnering up with the immune system? *Nat Rev Immunol* **22**, 576–588 (2022).

19. Qin, X. *et al.* CKLF instigates a "cold" microenvironment to promote MYCN-mediated tumor aggressiveness. *Science Advances* **10**, eadh9547 (2024).
20. Rickman, D. S., Schulte, J. H. & Eilers, M. The Expanding World of N-MYC-Driven Tumors. *Cancer Discov* **8**, 150–163 (2018).
21. Wei, J. S. *et al.* Clinically Relevant Cytotoxic Immune Cell Signatures and Clonal Expansion of T-Cell Receptors in High-Risk MYCN-Not-Amplified Human Neuroblastoma. *Clin Cancer Res* **24**, 5673–5684 (2018).
22. Tang, X. X. *et al.* High-Level Expression of EPHB6, EFNB2, and EFNB3 Is Associated with Low Tumor Stage and High TrkA Expression in Human Neuroblastomas. *Clin Cancer Res* **5**, 1491–1496 (1999).
23. Tang, X. X. *et al.* Implications of EPHB6, EFNB2, and EFNB3 expressions in human neuroblastoma. *Proceedings of the National Academy of Sciences* **97**, 10936–10941 (2000).
24. Yu, W. *et al.* Longitudinal single-cell multiomic atlas of high-risk neuroblastoma reveals chemotherapy-induced tumor microenvironment rewiring. *Nat Genet* **57**, 1142–1154 (2025).
25. Arora, R. *et al.* Spatial transcriptomics reveals distinct and conserved tumor core and edge architectures that predict survival and targeted therapy response. *Nat Commun* **14**, 5029 (2023).
26. Schulz, P. *et al.* Inducible re-expression of p16 in an orthotopic mouse model of pancreatic cancer inhibits lymphangiogenesis and lymphatic metastasis. *Br J Cancer* **99**, 110–117 (2008).

27. Takeuchi, H. *et al.* Loss of p16INK4a expression is associated with vascular endothelial growth factor expression in squamous cell carcinoma of the esophagus. *Int J Cancer* **109**, 483–490 (2004).

28. Zerrouqi, A., Pyrzynska, B., Febbraio, M., Brat, D. J. & Van Meir, E. G. P14ARF inhibits human glioblastoma-induced angiogenesis by upregulating the expression of TIMP3. *J Clin Invest* **122**, 1283–1295 (2012).

29. Kolev, V. *et al.* EGFR signalling as a negative regulator of Notch1 gene transcription and function in proliferating keratinocytes and cancer. *Nat Cell Biol* **10**, 902–911 (2008).

30. Kalyankrishna, S. & Grandis, J. R. Epidermal Growth Factor Receptor Biology in Head and Neck Cancer. *JCO* **24**, 2666–2672 (2006).

31. Wu, S. Z. *et al.* A single-cell and spatially resolved atlas of human breast cancers. *Nat Genet* **53**, 1334–1347 (2021).

32. Luo, J., Deng, M., Zhang, X. & Sun, X. ESICCC as a systematic computational framework for evaluation, selection, and integration of cell-cell communication inference methods. *Genome Res.* **33**, 1788–1805 (2023).

33. Wellenstein, M. D. & De Visser, K. E. Cancer-Cell-Intrinsic Mechanisms Shaping the Tumor Immune Landscape. *Immunity* **48**, 399–416 (2018).

34. Ponomarev, A., Gilazieva, Z., Solovyeva, V., Allegrucci, C. & Rizvanov, A. Intrinsic and Extrinsic Factors Impacting Cancer Stemness and Tumor Progression. *Cancers* (2022).

35. Akbay, E. A. *et al.* Activation of the PD-1 Pathway Contributes to Immune Escape

in EGFR-Driven Lung Tumors. *Cancer Discovery* **3**, 1355–1363 (2013).

36. Chen, N. *et al.* Upregulation of PD-L1 by EGFR Activation Mediates the Immune Escape in EGFR-Driven NSCLC: Implication for Optional Immune Targeted Therapy for NSCLC Patients with EGFR Mutation. *Journal of Thoracic Oncology* **10**, 910–923 (2015).
37. Angelova, M. *et al.* Evolution of Metastases in Space and Time under Immune Selection. *Cell* **175**, 751-765.e16 (2018).
38. Zhang, L. *et al.* Microenvironment-induced PTEN loss by exosomal microRNA primes brain metastasis outgrowth. *Nature* **527**, 100–104 (2015).
39. Sucker, A. *et al.* Acquired IFNγ resistance impairs anti-tumor immunity and gives rise to T-cell-resistant melanoma lesions. *Nat Commun* **8**, 15440 (2017).
